# Suppression of PP2A-B56α drives EMT in EGFR mutant non-small cell lung cancer

**DOI:** 10.1038/s41388-026-03772-2

**Published:** 2026-04-11

**Authors:** Brittany N. Heil, Garima Baral, Claire M. Pfeffer, Mei B. Bahler, Sanika S. Gulavani, Emily G. Smith, Lauren E. Gartenhaus, Anna K. Darling, Sydney J. Clifford, Whitney Smith-Kinnaman, Kasi Hansen, Emma H. Doud, Gaganpreet K. Mall, Aaron N. Hata, Nicole L. Anderson, Matthew R. Olson, Brittany L. Allen-Petersen

**Affiliations:** 1 Purdue University Interdisciplinary Life Sciences Program (PULSe), Purdue University, West Lafayette, IN USA; 2 Department of Biological Sciences, Purdue University, West Lafayette, IN USA; 3 Center for Proteome Analysis, Indiana University School of Medicine (IUSM), Indianapolis, IN USA; 4 Biochemistry and Molecular Biology, Indiana University School of Medicine (IUSM), Indianapolis, IN USA; 5 Massachusetts General Hospital Cancer Center, Boston, MA USA; 6 Department of Medicine, Massachusetts General Hospital and Harvard Medical School, Boston, MA USA; 7 Purdue Institute for Cancer Research, Purdue University, West Lafayette, IN USA

**Keywords:** Metastasis, Mechanisms of disease

## Abstract

Lung cancer is the leading cause of cancer-related deaths in the United States, and ~50% of these patients present with metastatic disease at diagnosis. Epithelial-to-Mesenchymal Transition (EMT) is an important initiating step in the metastatic cascade that allows cells to acquire the migratory and invasive phenotypes necessary for dissemination. The transcriptional reprogramming that takes place during EMT has been well described in multiple cancer types; however, the posttranslational regulatory mechanisms that govern EMT are poorly understood. Protein Phosphatase 2 A (PP2A) is serine/threonine (ser/thr) phosphatase that accounts for 50% of cellular ser/thr phosphatase activity and is critically important in regulating signaling homeostasis. PP2A dysregulation has been implicated in cell state regulation, EMT, and metastasis, but the roles of individual PP2A complexes are poorly understood. Our data indicate that suppression of the specific PP2A complex, PP2A-B56α, results in decreased expression of epithelial markers and increased expression of mesenchymal markers consistent with EMT. These molecular changes are associated with migratory and invasive phenotypes both in vitro and in vivo. Furthermore, these migratory phenotypes can be rescued with B56α overexpression. Together, these findings implicate B56α as a key regulator of cellular plasticity and highlight the dynamic nature by which PP2A-B56α posttranslationally regulates NSCLC EMT.

## Introduction

Lung cancer is the leading cause of cancer-related deaths worldwide and has an overall 5-year survival rate of approximately 26%, which plummets to just 9% with metastatic disease [1]. In Non-Small Cell Lung Cancer (NSCLC), the most common type of lung cancer, about 50% of patients have metastatic disease at the time of diagnosis [2]. During tumor progression, cancer cells acquire metastatic capabilities by undergoing an epithelial-to-mesenchymal transition (EMT). This unique form of cellular plasticity results in aggressive tumor phenotypes and is associated with broad therapeutic resistance [3–5]. Given the impact of EMT on tumor progression, understanding the mechanisms that regulate this process is essential for improving patient outcomes.

EMT is a dynamic and reversible process that is associated with reduced cell-cell junctions, acquired front-back polarity, and increased migratory and invasive capabilities, which allow cells to survive dissemination and travel to distant metastatic sites. Aberrant activation or expression of EMT-inducing transcription factors (EMT-TFs) (e.g., ZEB1, TWIST, and SNAIL) potently drives EMT in a wide variety of solid tumors [6–9]. In addition to these transcriptional programs, it is now appreciated that numerous posttranslational mechanisms play key roles in the regulation of EMT, including phosphorylation. Cancer cells can undergo EMT in response to a wide variety of signals, including therapeutic stress, without the need for additional oncogenic mutations, suggesting that posttranslational signaling mechanisms play a key role in driving EMT [4, 5]. Accordingly, several EMT-TFs are activated downstream of kinase signaling cascades, and direct phosphorylation of these transcription factors can impact their activity by altering both stability and subcellular localization [10]. Similarly, the localization of proteins involved in cellular adhesion is directly impacted by phosphorylation, suggesting that transcriptional and posttranslational regulatory mechanisms work together to maintain epithelial cell fates. These studies highlight the impact of non-genomic signaling mechanisms on cellular plasticity; however, our understanding of the posttranslational events that govern EMT remains limited. Additionally, as the analysis of patient tumors rarely includes posttranslational phosphorylation events, the dysregulation of posttranslational mechanisms may be underestimated in metastatic patient populations.

Protein phosphatases function as key gatekeepers of cellular plasticity, providing essential regulation of intracellular signaling cascades. Protein Phosphatase 2 A (PP2A) is a heterotrimeric serine-threonine phosphatase that has been identified as a vital tumor suppressor in many cancer types and negatively regulates many of the downstream effectors of the Epidermal Growth Factor Receptor (EGFR) pathway, a common oncogenic driver in NSCLC [11]. The PP2A holoenzyme is composed of 3 subunits: the scaffolding “A” subunit, the catalytic “C” subunit, and 16 regulatory “B” subunits that determine substrate specificity. PP2A has been implicated in the direct regulation of many cell-cell junction proteins, including desmosomes, E-cadherin, and β-catenin [12]. Pharmacological inhibition or genetic loss of total PP2A activity disrupts these junctions and contributes to metastasis in several cancer types; however, the contribution of individual B subunits to the regulation of cancer cell signaling pathways is largely unknown. Using shRNA knockdown screens, Sablina et al. determined that the specific PP2A subunit, B56α, displays potent tumor suppressor capabilities [13]. Similarly, genetic loss of B56α results in low-penetrance tumor formation and increased stem-like properties after long latency [14]. In melanoma patients, expression of B56α is significantly reduced in metastatic versus primary tumors, implicating a role for B56α in tumor dissemination [15]. Recently, our lab demonstrated that loss of B56α in combination with oncogenic KRAS^G12D^ expression increased chromatin accessibility at EMT-related genes and exacerbated cancer-associated cell fate transitions [16]. Together, these findings suggest that PP2A-B56α functions as a key regulator of cancer cell plasticity; however, the contribution of this B subunit to NSCLC EMT has not been assessed. Therefore, we hypothesize that PP2A-B56α inhibits NSCLC cancer cellular plasticity and suppresses the posttranslational activation of EMT signaling programs.

Here, we show that reduced B56α expression correlates with decreased overall NSCLC patient survival. In primary EGFR-mutant NSCLC patient-derived cell lines, B56α expression is correlated with expression of the epithelial cell marker, E-cadherin. Similarly, suppression of B56α results in a morphological shift from an epithelial to a mesenchymal cell state consistent with EMT. This cell state change is associated with a loss of epithelial cell-cell junctions and a corresponding increase in mesenchymal marker expression. Beyond these markers, the B56α-dependent EMT phenotype is associated with large-scale proteomic and phosphoproteomic changes indicative of broad cellular rewiring and aberrant pathway activation. In line with the role of EMT in metastasis, B56α knockdown cells have a significant increase in migratory and invasive capacity both in vitro and in vivo. These phenotypes can be rescued with overexpression of B56α, highlighting the dynamic and reversible nature of this cellular plasticity. Together, our findings support a critical role for PP2A-B56α in the regulation of EMT cell state dynamics and metastasis in EGFR-driven NSCLC.

## Materials and methods

### Cell culture

All primary and established NSCLC cell lines were cultured in RPMI-1640 (Fisher SH3002701) containing 10% fetal bovine serum (Fisher FB12999102). Primary cell lines were previously established from patients following core biopsy or pleural effusion as previously described [17, 18]. Patients signed informed consent to give permission for research to be completed using their samples through a Dana-Farber-Harvard Cancer Center Institutional Review Board-approved protocol. To generate B56α overexpression cells, B56α was cloned from the pCEP-4HA B56α plasmid (Addgene #14532) into a modified pSIN-PURO or pINDUCER-20 (Addgene #109334) vector [19]. Lentivirus was then used to establish stable HA-tagged B56α overexpression and shRNA B56α knockdown cell lines. Lentivirus was produced using HEK293T cells transfected using Lipofectamine 3000 (Fisher Scientific, L3000015), the plasmid of interest, and packaging plasmids (pAX.2 Addgene Plasmid #12260 and pMD2.G Addgene Plasmid #12259). Parental cell lines were transduced with viral media containing the plasmid of interest (pSIN or SMARTvector shRNA plasmids; VSC11709 (shSCR puromycin), V3SH11240-225202507 (shB56α1), V3SH11240-229314505 (shB56α2), Horizon Discovery or VB010000-9537kcx (shSCR blasticidin), VB900196-7436ebe (shZeb1), VB900196-7435juy (shB55α1), VB900196-7437rce (shB55α2) and selected with the appropriate antibiotic. For shZeb1 studies, cells were transduced for 96 h. All cell lines were routinely tested for Mycoplasma using PCR-based strategies and grown at 37 °C in 5% CO_2_ atmosphere. For inducible B56α experiments, cells were cultured in 500 ng/mL of Doxycycline. For B56α rescue experiments, cells were seeded and transiently transfected with HA-tagged B56α using Lipofectamine 3000 (Fisher Scientific, L3000015) according to manufacturer instructions.

### Quantitative RT-PCR

RNA was isolated using the ThermoScientific GeneJet RNA Purification Kit (Thermofisher, #K0732). cDNA was generated using the High-Capacity cDNA Reverse Transcription Kit (Fisher Scientific, #43-688-14). Quantitative RT-PCR was performed using PowerUp SYBR Green Master Mix reagent (Fisher Scientific, #A25743) on the QuantStudio 3 with the indicated primers (Supplementary Methods Table 1). The fold change relative to vehicle/control was analyzed using the ΔΔ(C_t_) method.

### Immunoprecipitation (IP)

HCC827 EV and B56αOE cells were lysed with IP lysis buffer (14321D, Invitrogen) supplemented with protease and phosphatase inhibitor cocktail (PIA32959, Thermo Scientific). IgG (sc-2025, Santa Cruz Biotechnology) or HA (2367S, Cell Signaling Technology) antibodies were conjugated with Dynabeads protein-G (10007D, Invitrogen) for pulldown, and IP was conducted as per the manufacturer’s protocol. Following the IP, beads were washed and eluted in 2.5X SDS buffer and boiled for 10 min at 95° C. The protein samples were then run on an SDS-gel, transferred, and processed as mentioned below.

### Western blotting

Cells were lysed (20 mM Tris, pH 7.5, 50 mM NaCl, 0.5% Triton X-100, 0.5% deoxycholate, 0.5% SDS, and 1 mM EDTA) and protein concentration was determined using the DC protein assay kit (Bio-Rad, 5000112). Lysate was prepared using XT Sample Buffer (Bio-Rad, 1610791) and XT Reducing Agent (Bio-Rad, 1610792), then boiled at 95 °C. SDS-PAGE was run on 4-12% gradient bis-tris protein gel (Bio-Rad, 3450123) and transferred to PVDF membrane. Membrane was blocked in Licor TBS blocking buffer (Fisher Scientific, NC1660550) for 1 h at room temperature and incubated with primary antibody overnight at 4 °C (Supplementary Methods Table 2) and then incubated with secondary antibody for 1 h at room temperature. Membranes were scanned using the Licor Odyssey DLx Imaging System and analyzed using Image Studio software v5.2.5. For drug treatment studies, cells were seeded in 6-well plates, and Osimertinib (Medchem 1421373-65-0, Lot 273365) was immediately added to the culture upon plating. After 24 h, cells were lysed and processed for western blot analysis.

### Immunocytochemistry

Cells were seeded on top of sterile glass coverslips in 24-well cell culture plates. After 48 h, cells were fixed in 4% paraformaldehyde for 20 min at room temperature. Cells were permeabilized by 40μg/mL digitonin (Sigma Aldrich, D141-100MG) for 1 min. Slides were incubated at 4°C overnight in primary antibody solutions (diluted with 2% bovine serum albumin (BSA) in PBS). Slides were incubated in secondary antibody solutions (diluted in 2% BSA in PBS) for 1 h at room temperature and DAPI stained for 5 min at room temperature (Supplementary Methods Table 3). Coverslips were added to slides using ProLong Gold anti-fade reagent (Invitrogen, P36934). All rinses between solutions were done with PBS-T (phosphate-buffered saline and 0.05% Tween-20). Slides were imaged with a Nikon Ni-U Upright Microscope with epifluorescence and processed with FIJI software v2.14.0.

### Transwell migration assay

Transwell inserts were soaked in serum-free RPMI-1640 media for one h prior to seeding cells. Cells were seeded at a density of 2 × 10^4^ per well in serum-free RPMI-1640 with RPMI-1640 containing 10% FBS in the lower chamber. After incubating for 48 h, or 24 h for rescue experiments, the media was aspirated and stained with crystal violet for 1.5 h. Migrated cells were imaged with the EVOS M7000 and quantified with FIJI software.

### Spheroid invasion assay

50,000 cells per well were plated in 96-Well Clear Ultra Low Attachment Microplates (Corning, 07-201-680). After 72 h, spheroids were removed from low attachment plates and embedded by plating on top of 80% Matrigel (Corning 356231, lot: 2024001) and suspended in 10% Matrigel. Spheroids were imaged every 24 h for 96 h, at which point spheroids were fixed and processed for immunofluorescent imaging. Spheroid circularity was calculated using FIJI software v2.14.0.

### Proliferation assays

Proliferation: 100,000 cells were plated in triplicate in 4, 6-well plates. Cell number was quantified every 24 h. Population doubling: 100,000 cells were plated in triplicate into 6-well plates. Every 72 h, cells were trypsinized and cell number was quantified. Cells were then passaged by replating 100,000 cells onto a new plate. Cells were quantified over a total of 12 days, and population doubling was calculated using the natural log.

### Phosphoproteomics

Sample preparation, mass spectrometry analysis, bioinformatics, and data evaluation for quantitative proteomics experiments were performed in collaboration with the Indiana University Proteomics Center for Proteome Analysis at the Indiana University School of Medicine, similarly to previously published protocols [20, 21].

*Protein Extraction and Digestion*—Cell pellets were resuspended in 100 µL 8 M Urea, 100 mM Tris, pH 8.5. The resuspended cell pellets were transferred to Diagenode Bioruptor tubes (Cat No: C30010010). Cells were lysed via Diagenode Bioruptor, 30 s on/30 s off, for 30 cycles. Samples were then clarified by centrifuging for 30 min at 12,000 rcf. Supernatants were analyzed in a Bradford assay (Biorad Cat No: 5000002) to determine protein concentration. 1 mg of each sample was treated with 5 mM tris (2-carboxyethyl) phosphine hydrochloride (Sigma-Aldrich Cat No: C4706) to reduce disulfide bonds, and the resulting free cysteine thiols were alkylated with 10 mM chloroacetamide (Sigma Aldrich Cat No: C0267). Samples were diluted with 50 mM Tris.HCl pH 8.5 (Sigma-Aldrich Cat No: 10812846001) to a final urea concentration of 2 M for overnight Trypsin/Lys-C digestion at 35 °C (1:25 protease: substrate ratio, Mass Spectrometry grade, Promega Corporation, Cat No: V5072).

*Peptide Purification and phosphopeptide enrichment*—Digestions were quenched with trifluoroacetic acid (TFA, 0.5% v/v) and desalted on Waters Sep-Pak® Vac cartridges (Waters™ Cat No: WAT054955) with a wash of 1 mL 0.1% TFA followed by elution in 3 × 0.2 mL of 70% acetonitrile 0.1% formic acid (FA). Peptides were dried by speed vacuum. Samples were resuspended in phosphopeptide binding buffer, and phosphopeptides were enriched using Thermo Fisher Scientific High Select TiO2 tips (Cat No A32993) according to the manufacturer’s instructions. Flow through (non-phosphopeptides) and phosphopeptides were dried down by speed vacuum.

*TMTpro labeling*—An equivalent of 50 μg of the global (non-phospho) peptides and all of each phosphopeptide enrichment were resuspended in 100 mM triethylammonium bicarbonate (TEAB, pH 8.5 from 1 M stock). Each sample was then labeled overnight at room temperature, with 0.5mg of Tandem Mass Tag Pro (TMTpro™) reagent (16-plex kit, manufacturer's instructions, Thermo Fisher Scientific, TMTpro™ Isobaric Label Reagent Set; Cat No: 44520, lot no. ZA382395, see Table X below). Reactions were quenched with 0.3% hydroxylamine (v/v) at room temperature for 15 min. Labeled peptides were then mixed and dried by speed vacuum.

*High pH Basic Fractionation*—Half of the combined global sample and all of the phosphopeptide sample were resuspended in 0.5% TFA and fractionated on a Waters Sep-Pak® Vac cartridge (Waters™ Cat No: WAT054955) with a 1 mL wash of water, 1 mL wash of 5% acetonitrile, 0.1% triethylamine (TEA) followed by elution for the global sample in 8 fractions of 12.5%, 15%, 17.5%, 20%, 22.5%, 25%, 30%, and 70% acetonitrile, all with 0.1% TEA).

*Nano-LC-MS/MS*—Mass spectrometry was performed utilizing an EASY-nLC 1200 HPLC system (SCR: 014993, Thermo Fisher Scientific) coupled to an Eclipse™ mass spectrometer with FAIMSpro interface (Thermo Fisher Scientific). Each multiplex was run on a 25 cm Aurora Ultimate TS column (Ion Opticks Cat No: AUR3-25075C18) in a 50 °C column oven with a 180-min gradient. For each fraction, 2% of the sample was loaded and run at 350 nl/min with a gradient of 8-38%B over 98 min; 30-80% B over 10 mins; held at 80% for 2 min; and dropping from 80-4% B over the final 5 min (Mobile phases A: 0.1% formic acid (FA), water; B: 0.1% FA, 80% Acetonitrile (Thermo Fisher Scientific Cat No: LS122500)). The mass spectrometer was operated in positive ion mode, default charge state of 2, advanced peak determination on, and lock mass of 445.12003. Three FAIMS CVs were utilized (-45 CV; -55 CV; -65CV), each with a cycle time of 1 s and with identical MS and MS2 parameters. Precursor scans (m/z 400-1600) were done with an Orbitrap resolution of 120000, RF lens% 30, 50 ms maximum inject time, standard automatic gain control (AGC) target, minimum MS2 intensity threshold of 2.5e4, MIPS mode to peptide, including charges of 2 to 6 for fragmentation with 60 sec dynamic exclusion shared across the cycles, excluding isotopes. MS2 scans were performed with a quadrupole isolation window of 0.7 m/z, 34% HCD collision energy, 50000 resolution, 200% AGC target, dynamic maximum IT and fixed first mass of 100 m/z.

*Mass spectrometry Data Analysis*—Resulting RAW files were analyzed in Proteome Discover™ 2.5.0.400 (Thermo Fisher Scientific) [22] with a *Mus musculus* UniProt reference proteome FASTA (downloaded 051322) plus common laboratory contaminants (73 sequences). SEQUEST HT searches were conducted with full trypsin digest, a maximum of 3 missed cleavages; precursor mass tolerance of 10 ppm, and a fragment mass tolerance of 0.02 Da. Static modifications used for the search were: (1) TMTpro label on peptide N-termini, (2) TMTpro label on lysine (K), and (3) carbamidomethylation on cysteine (C) residues. Dynamic modifications used for the search were (1) oxidation on M, (2) phosphorylation on S, T or Y, (3) acetylation on protein N-termini, (4) methionine loss on protein N-termini or (5) acetylation with methionine loss on protein N-termini. A maximum of 3 dynamic modifications was allowed per peptide. Percolator False Discovery Rate was set to a strict setting of 0.01 and a relaxed setting of 0.05. IMP-ptm-RS node was used for all modification site localization scores. Values from both unique and razor peptides were used for quantification. In the consensus workflows, peptides were normalized by total peptide amount with no scaling. Unique and razor peptides were used, and all peptides were used for protein normalization and roll-up. Quantification methods utilized TMTpro isotopic impurity levels available from Thermo Fisher Scientific. Reporter ion quantification filters were set to an average S/N threshold of 5 and a co-isolation threshold of 30%. Resulting grouped abundance values for each sample type, abundance ratio values and respective p-values (Protein Abundance based on ANOVA, individual protein based) from Proteome Discover were exported to Microsoft Excel. Pathway enrichment was performed using the online tool Metascape v3.5 (http://metascape.org) [23].

### Tail vein injection mouse study

All animal studies were completed in compliance with Purdue University (West Lafayette, IN) animal care and use guidelines after approval by the Purdue Institutional Animal Care and Use Committee. NOD.Cg-Rag1^tm1Mom^ Il2rg^tm1Wjl^/SzJ (NRG) mice (JAX 007799) were used for the tail vein injection study. Mice were injected intravenously with 2 million H1650 cells in 100μL PBS. At 6 weeks post-injection, mice were euthanized by CO_2_ followed by cervical dislocation. Lung and liver were removed from each mouse, perfused and inflated with PBS/heparin, and then fixed in 10% neutral buffered formalin and paraffin-embedded.

### H&E tissue staining and immunofluorescence (IF)

For both H&E and IF, lung and liver tissues were sectioned at 6 μm thickness using a Thermo HM355S microtome. Slides were baked overnight at 55 °C and H&E staining was performed as previously described [16]. For IF, slides were rehydrated, and double antigen retrieval was used (high pH cat:00-4956-58; low pH cat:00-4955-58). Slides were blocked with 3% BSA in PBS for 1 h at room temperature. Primary antibodies were used as indicated and incubated overnight at 4 °C (Supplementary Table 3). Tissues were washed with PBS-T and incubated in secondary antibodies for 1 h, and DAPI stained for 5 min at room temperature. Cells were mounted with Prolong Gold mounting medium and imaged on a Nikon Ni-U Upright Microscope.

### Statistical considerations

All experiments were performed in at least three independent biological replicates, and graphs are plotted with standard deviation (s.d.) unless otherwise denoted in the figure legend. Statistical significance was determined using a two-tailed Student’s *t* test (2 samples) or a one-way ANOVA with post hoc analysis using GraphPad Prism (GraphPad Software)(3 samples or more). If experimental results displayed unequal variance by Bartlett’s test, then a Brown-Forsythe and Welch’s one-way ANOVA test was performed. Values less than *p* < 0.05 were considered significant. *p < 0.05, **p < 0.01, ***p < 0.001, ****p < *0.0001.*

## Results

### PP2A-B56α plays a critical role in NSCLC tumor progression

**Fig. 1:**
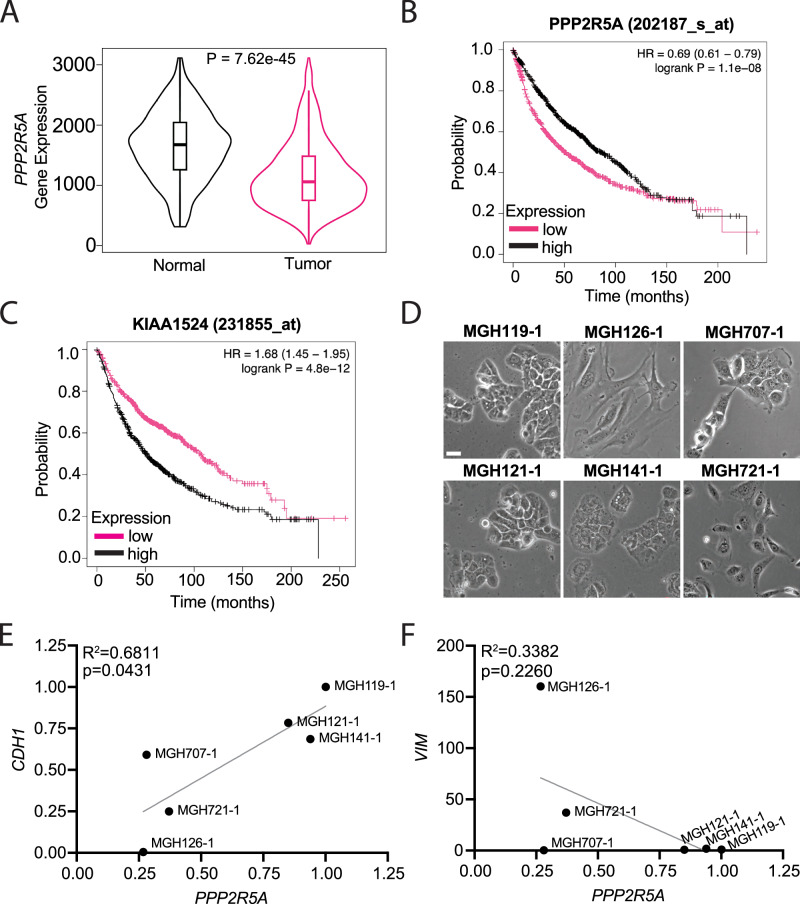
Low B56α expression correlates with poor prognosis in NSCLC. **A** Normal and tumor tissue from lung cancer patient analyzed by RNA sequencing for B56α (*PPP2R5A*) expression, showing decreased expression in tumor tissue compared to normal adjacent tissue. **B** Survival curve of lung cancer patients with low or high expression of B56α, with low expression of B56α correlating with decreased survival. **C** Survival curve of lung cancer patients with low or high expression of CIP2A (*KIAA1524*), with high expression of CIP2A correlating with decreased survival. KMPlotter was used to analyze (**A**–**C**). **D** Representative brightfield images of primary patient cell lines from NSCLC patients (scale bar = 100 μm). **E** Correlation of the mRNA expression of E-cadherin (*CDH1*) and B56α in a panel of NSCLC primary patient cell lines (*n* = 3 biological replicates). **F** Correlation of the mRNA expression of Vimentin (*VIM*) and B56α in a panel of NSCLC primary patient cell lines (*n* = 3 biological replicates).

To identify the relationship between PP2A-B56α expression and NSCLC patient outcomes, we analyzed publicly available genomic data from NSCLC patients. We found that B56α (*PPP2R5A*) mRNA expression is significantly decreased in NSCLC tumors compared to adjacent normal tissue (Fig. 1A). Similarly, low B56α (*PPP2R5A*) mRNA expression or high mRNA expression of Cancerous Inhibitor of PP2A (CIP2A, *KIAA1524*), an endogenous inhibitor of the B56 family of subunits, correlated with decreased overall patient survival (Fig. 1B, C) [24]. We and others have previously shown that B56α directly dephosphorylates the oncoprotein, c-MYC (MYC), at Serine62 (S62), which drives MYC degradation [16, 25–28]. Recently, we established that activation of the mitogen-activated protein kinase (MAPK) pathway downstream of EGFR/RAS signaling leads to increased CIP2A expression, decreased PP2A-B56α activity, and increased phosphorylation of S62 MYC [16]. These signaling changes were associated with increased cellular plasticity and EMT gene programs. Based on these findings, we sought to determine the expression levels of B56α, E-cadherin (epithelial marker), and Vimentin (Mesenchymal marker) in primary NSCLC patient-derived cell lines. These lines were generated from patients with NSCLCs harboring *EGFR* exon 19 deletions (activating alteration) either before or after treatment with EGFR tyrosine kinase inhibitors (Fig. 1D) [17, 18]. The lines exhibited diverse morphological characteristics ranging from epithelial to mesenchymal, and had varied expression of E-cadherin and Vimentin. Using quantitative RT-PCR (qRT-PCR) we determined that the expression B56α is positively correlated with E-cadherin (*p* < *0.05*)(Fig. 1E) and trended with decreased Vimentin expression (*p* = *0.226*)(Fig. 1F), suggesting that PP2A-B56α may function to sustain an epithelial cell identity in NSCLC.

### PP2A-B56α suppression results in morphological and molecular changes associated with EMT

**Fig. 2:**
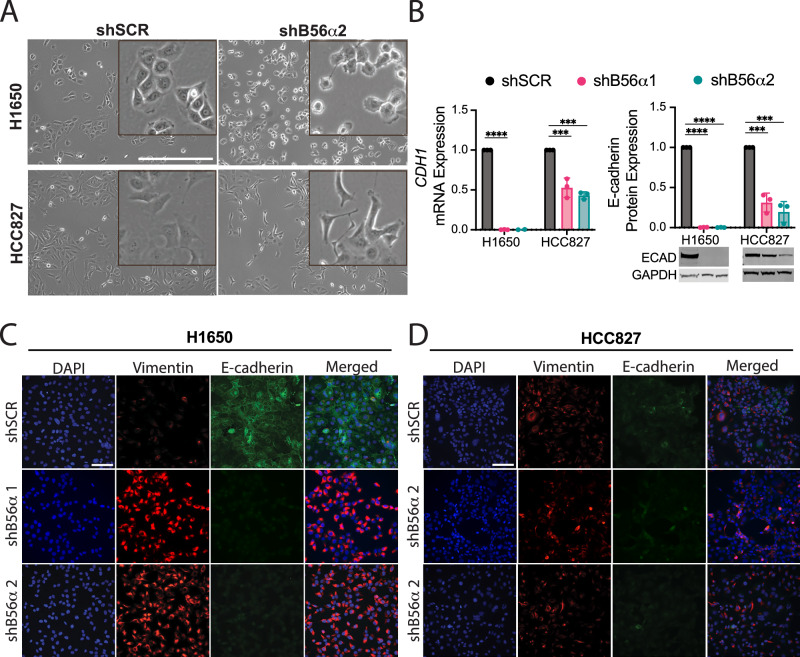
Knockdown of B56α drives EMT marker expression. **A** Representative brightfield images of H1650 and HCC827 cell lines with shRNA-mediated knockdown of B56α (KD) compared to shSCR control (scale bar=500 μm). Inset denotes digital zoom. **B** mRNA and protein expression of E-cadherin in H1650 and HCC827 KD cell lines compared to shSCR control (*n* = 3 biological replicates) **C** Representative immunofluorescent images from three biological replicates of H1650 KD and **D** HCC827 KD cell lines with DAPI, Vimentin, and E-cadherin compared to shSCR control (scale bar = 100 μm).

To determine the impact of PP2A-B56α suppression on NSCLC tumor phenotypes, we first knocked down the B56α subunit of PP2A using two independent shRNAs (shB56α1 and shB56α2) in human NSCLC cell lines with EGFR exon 19 deletion (EGFR del19) (Supplementary Fig. 1A, B). Both H1650 and HCC827 shB56α cells exhibited decreased epithelial “cobblestone” morphology and increased front-back polarity compared to a scrambled shRNA control (shSCR) (Fig. 2A and Supplementary Fig. 1C). To determine if these morphological changes were associated with a loss in epithelial identity, expression of E-cadherin and Vimentin was analyzed in shB56α cells. B56α knockdown resulted in a significant decrease in E-cadherin mRNA and protein expression compared to shSCR controls (Fig. 2B). H1650 and HCC827 shB56α cells were then fixed, and DAPI, Vimentin, and E-cadherin were analyzed by immunofluorescent (IF) imaging. H1650 shB56α cells displayed a drastic loss of E-cadherin with a corresponding increase in Vimentin expression (Fig. 2C). Similar results were found HCC827 shB56α cells albeit to a lesser degree, potentially indicative of a partial EMT phenotype (Fig. 2D). In contrast, knockdown of the PP2A B55α subunit (shB55α) did not result in any morphological changes or altered expression of E-cadherin or Vimentin, suggesting that this phenotype may be specific to B56α (Supplementary Fig. 2A–D). Together, these results suggest that PP2A-B56α functions to maintain NSCLC epithelial identity, with inhibition of PP2A-B56α promoting a mesenchymal cell state.

### Loss of PP2A-B56α exacerbates tumorigenic phenotypes in NSCLC cell lines

**Fig. 3:**
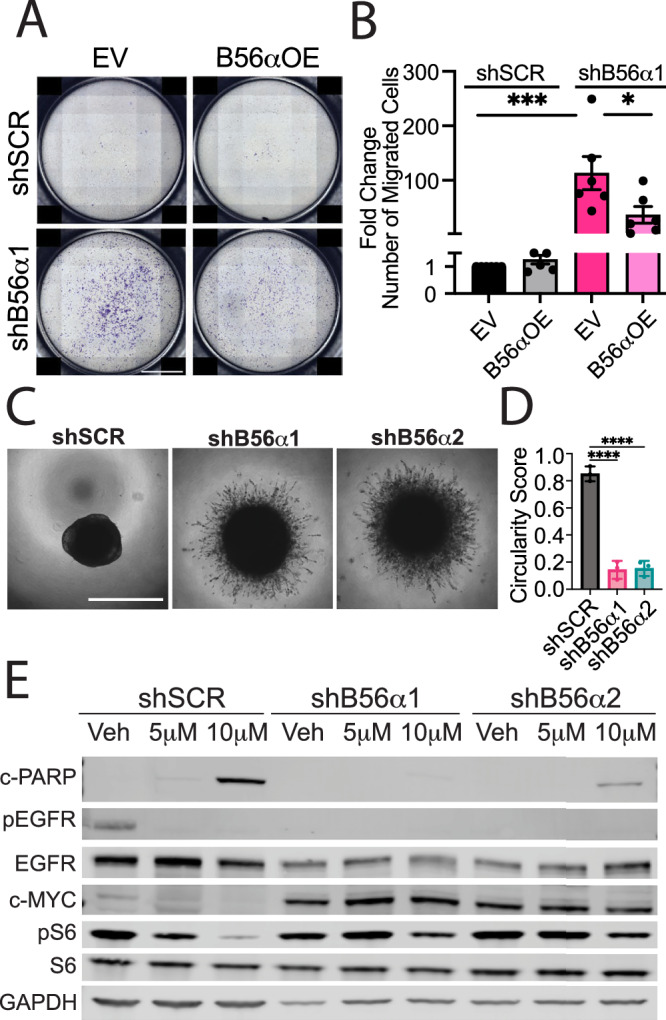
Suppression of B56α increases invasive NSCLC phenotypes. **A** Representative full-well images of trans-well migration assay of H1650 KD cells compared to shSCR control, with transient overexpression of B56α (B56αOE) compared to empty vector control (EV). Scale bar = 2 mm. **B** Quantification of panel A showing the fold change in average number of migrated cells per well (*n* = 6 biological replicates). **C** Representative images of H1650 KD spheroids at 96 h after embedding in Matrigel (scale bar = 1 mm). **D** Quantification of D using circularity score. **E** Representative western blot images of H1650 KD cells treated with 5μM or 10μM Osimertinib for 24 h showing cleaved PARP (c-PARP), phospho-EGFR (p-EGFR; Y1068), total EGFR, c-MYC, phospho-S6 (pS6; S240/244), total S6, and GAPDH compared to vehicle (DMSO) control.

The process of EMT underlies metastatic phenotypes. Therefore, to determine if the B56α-mediated molecular changes are associated with increased migratory capacity, cells were plated in a trans-well migration assay. H1650 shSCR and shB56α cells were plated and allowed to migrate for 24 h prior to fixing and staining with crystal violet for quantification. Suppression of PP2A-B56α significantly increased the number of migratory cells compared to shSCR control (Fig. 3A). To assess if this phenotype is specific to B56α, we transiently overexpressed exogenous B56α (B56αOE) in shB56α cells. Restoration of B56α expression attenuated the migratory capacity of shB56α cells, reducing migration by 30% compared to empty vector control (EV) (Fig. 3A, B and Supplementary Fig. 3A). To determine the impact of PP2A inhibition on the invasive capacity of H1650 cells, shSCR and shB56α cells were first plated into low attachment round bottom plates to form spheroids and then embedded into media supplemented with 10% Matrigel and imaged over time (96 h). H1650 shSCR cells formed well-organized structures with defined borders and had minimal cell invasion (Fig. 3C and Supplementary Fig. 3B). In stark contrast, shB56α spheroids were highly invasive with no defined border, as evidenced by an 80% decrease in circularity score where 1 is a perfect circle (Fig. 3C, D). Additionally, these structures had an abundance of cells (both single cells and clusters) that invaded into the surrounding extracellular matrix. Similar to growth in 2D cell culture (Fig. 2), these invasive cells expressed high levels of the mesenchymal marker, Vimentin (Supplementary Fig. 3C).

To determine if the shift towards a mesenchymal cell state in response to B56α inhibition is associated with a loss of dependency on EGFR signaling, H1650 shB56α cell lines were treated with increasing doses of the EGFR inhibitor Osimertinib for 24 h and then subjected to Western Blot analysis. Drug doses were chosen based on the published H1650 Osimertinib IC50 of 2.5μM (DepMap) after 72 h [29]. In shSCR control cells, Osimertinib treatment reduced EGFR phosphorylation at Tyrosine1068 (pY1068), a marker of EGFR activation, and induced the cleavage of poly (ADP-ribose) polymerase (PARP), a marker of apoptosis (Fig. 3E). In contrast, suppression of B56α resulted in an almost complete loss of EGFR pY1068 levels at baseline in vehicle-treated conditions. To determine if B56α expression can rescue the loss of pY1068 EGFR, H1650 shB56α cells were transduced to express an inducible B56α and analyzed for signaling. After 21 days of culturing with or without doxycycline (DOX), we found no significant changes in E-cadherin or Vimentin; However, pY1068 was increased in +DOX conditions, suggesting that B56α may be indirectly contributing to the regulation of phosphorylation at this site (Supplementary Fig. 3E, F). Paradoxically, despite the loss of phospho-EGFR activation, shB56α cells displayed increased proliferation rates (Supplementary Fig. 3D), potentially indicating that these cells have become uncoupled from EGFR signaling pathways. Consistent with EGFR-independent survival, the induction of apoptosis (cleaved-PARP) in response to Osimertinib was attenuated in shB56α cells compared to shSCR controls (Fig. 3E). Previous studies have determined that cytotoxic responses to EGFR inhibitors are mediated in part through decreased CIP2A expression and increased PP2A activity [30, 31]. However, we found that Osimertinib treatment decreased CIP2A expression in all conditions, but was only effective at killing B56α wild-type cells. Previous studies have shown that B56α and B55α display differential functions in the regulation of MYC, with B56α leading to MYC degradation and B55α contributing to MYC activity. In shB56α cells, both MYC expression and a MYC-mediated pathway, phospho-S6 Kinase, were dramatically increased in response to B56α knockdown and remained high even in the presence of Osimertinib (Fig. 3E). Consistent with the differential roles of these B subunits, knockdown of B55α had no impact on MYC expression (Supplementary Fig. 2). These results highlight a crucial role for B56α in regulating invasive NSCLC phenotypes downstream of EGFR activation, potentially through the activation of MYC-dependent pathways.

### Overexpression of PP2A-B56α drives an epithelial cell state and suppresses tumor phenotypes

**Fig. 4:**
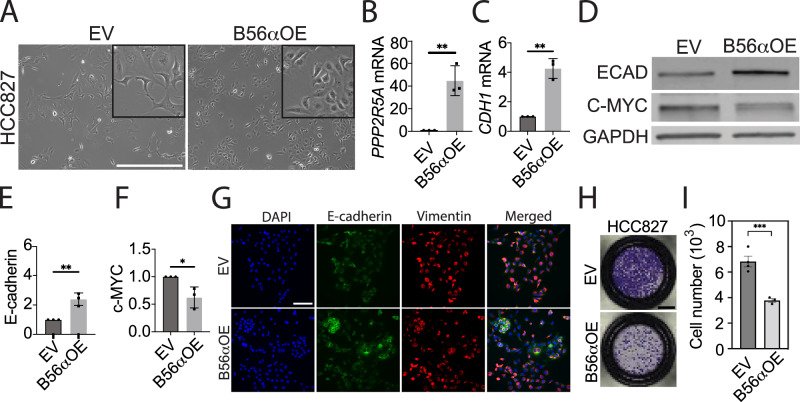
Overexpression of B56α increases E-cadherin expression and reduces migratory capacity. **A** Representative brightfield images of HCC827 stable overexpression (B56αOE) compared to empty vector control (EV). Scale bar = 500 μm. **B** mRNA expression of B56α (*PPP2R5A*) and **C** E-cadherin (*CDH1*) in HCC827 B56αOE cells compared to EV. **D** Representative western blot of E-cadherin, c-MYC, and GAPDH protein expression in HCC827 B56αOE compared to EV. **E** Quantification of E-cadherin protein and **F** c-MYC protein from western blot in (**D**) (*n* = 3 biological replicates). **G** Representative images of HCC827 B56αOE compared to EV showing DAPI, E-cadherin, and Vimentin by immunofluorescent imaging from three biological replicates (scale bar = 100 μm). **H** Representative full-well images of transwell migration assay of HCC827 B56αOE cells compared to EV from three biological replicates. **I** Quantification of number of cells migrated in panel H (scale bar=2 mm).

To determine if increased expression of PP2A-B56α shifts cells towards an epithelial cell fate, we analyzed NSCLC cell lines with stable overexpression of PP2A-B56α. HCC827 and H1650 cells were transduced with either empty vector (EV) or HA-tagged B56α to generate stable overexpression lines (B56αOE). Exogenous B56α co-immunoprecipitated with both the PP2A A and C subunits, indicating the formation of an active complex (Supplementary Fig. 4A). Consistent with the dramatic morphological changes that occur with suppression of B56α, H1650 was highly intolerant of B56α overexpression and senesced shortly after transduction (Supplementary Fig. 4B). PP2A-B56α stable overexpressed in HCC827 resulted in cells shifting to a more epithelial “cobblestone” morphology (Fig. 4A, B), which was accompanied by increased expression of E-cadherin as shown by qRT-PCR and western blot (Fig. 4C–E). Protein expression of the PP2A-B56α target, MYC, was significantly decreased with B56αOE, indicating that PP2A is active in these cells (Fig. 4D, F). Analysis of B56αOE cells by immunofluorescence identified increased E-cadherin-positive cell clusters compared to EV control cells. In contrast, there was no significant loss of Vimentin expression, suggesting that B56α overexpression increases epithelial signaling programs (Fig. 4G). In addition to increased E-cadherin expression, B56αOE cells were significantly less migratory as shown by a trans-well migration assay compared to EV (Fig. 4H, I). Collectively, these findings suggest that low B56α expression increases the propensity of NSCLC cells to undergo cell state changes, while high B56α expression reinforces a more restricted epithelial cell state.

### Suppression of B56α results in cellular rewiring with large-scale proteomic changes

**Fig. 5:**
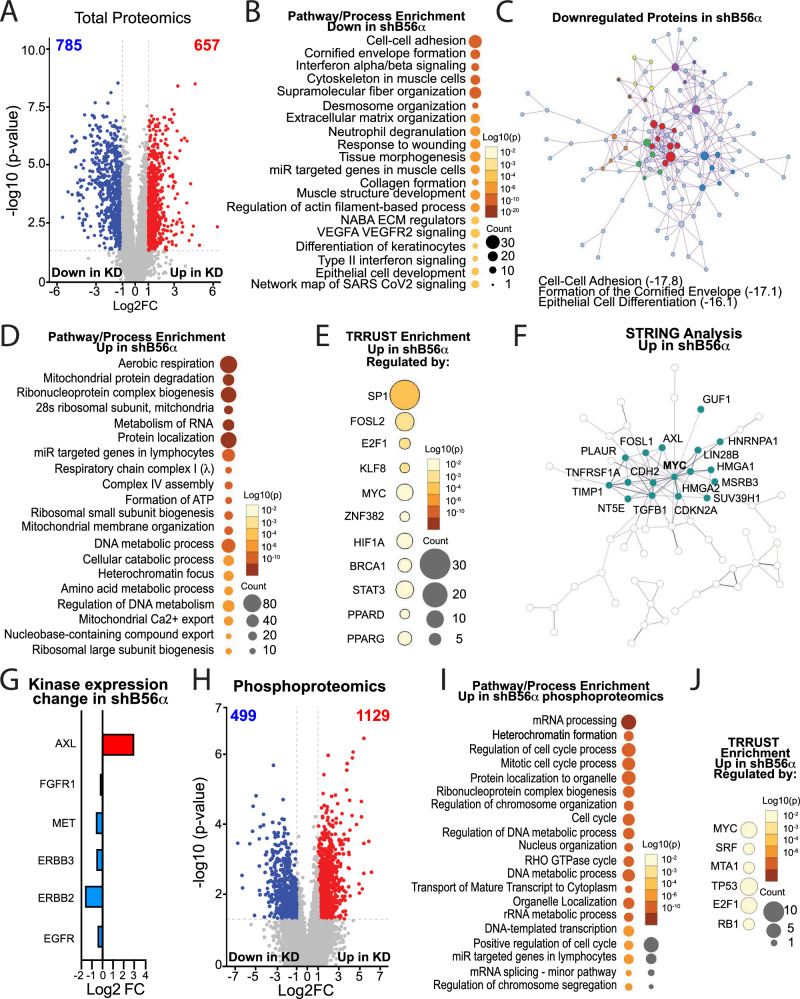
B56α knockdown alters the expression of a diverse set of proteins involved in tumorigenic plasticity. **A** volcano plot of total proteomic changes in H1650 KD cells compared to shSCR control (Log_2_FC <-1 or >1; *p* < 0.05) **B** Pathway and Process Enrichment Analysis using Metascape analysis of significantly downregulated proteins in total proteomics (Log_2_FC <-2, *p* < 0.05). **C** MCODE Metascape analysis of pathway enrichment with protein interactions of significantly downregulated proteins in total proteomics (Log_2_FC <-2, *p* < 0.05). Top 3 enrichment pathways listed with Log_2_ p-value in parentheses. **D** Metascape Pathway and Process Enrichment Analysis of significantly upregulated proteins in total proteomics (Log_2_FC > 1, *p* < 0.05). **E** TRRUST enrichment analysis using Metascape, identifying gene sets that are enriched in shB56α and regulated by specific transcription factors. **F** STRING analysis of the top 15% upregulated proteins in shB56α, highlighting the enriched c-MYC-centered network. **G** Curated list of kinases significantly changed in shB56α total proteomics. **H** Volcano plot of phosphoproteomic changes in H1650 shB56α (Log_2_FC <-1 or >1; *p* < 0.05). **I** Metascape Pathway and Process Enrichment Analysis of significantly upregulated proteins in phosphoproteomics (Log_2_FC > 2, *p* < 0.05). J) TRRUST enrichment analysis using Metascape, identifying gene sets that are enriched in shB56α phosphoproteomics and regulated by specific transcription factors.

As knockdown of B56α resulted in dramatic morphological and signaling changes (Figs. 2 and 3), we performed global proteomics and phosphoproteomics on H1650 shSCR and shB56α cells to understand the impact of B56α phosphatase inhibition on the NSCLC proteome. H1650 shSCR and shB56α were plated in triplicate and harvested after 48 h. Proteins were Tandem Mass Tag labeled and analyzed by mass spectrometry using Nano-LC-MS/MS as outlined in Materials and Methods. Proteomic analysis revealed that suppression of B56α resulted in 1,442 significantly changed total proteins, with 785 downregulated and 657 upregulated (Fig. 5A and Supplementary Data Table 1). Consistent with a loss of epithelial characteristics, Gene Ontology (GO) enrichment identified several cellular adhesion and cell junction signaling programs as being significantly downregulated in shB56α cells compared to shSCR controls (Fig. 5B and Supplementary Data Table 2). Analysis of transcription factor target interactions using TRRUST [32] revealed a decrease in ZEB1-regulated targets, including epithelial proteins such as E-cadherin and EpCAM, in shB56α cells (Supplementary Fig. 5A). Given these results, we quantified the expression of classical EMT-TFs, including *ZEB1*, SNAIL (*SNAI1*), SLUG (*SNAI2*), and TWIST (*TWIST1*). *ZEB1* mRNA expression was increased in shB56α cells, albeit in a variable manner, whereas *SNAI1*, *SNAI2*, and *TWIST* mRNA expression were decreased comparatively to shSCR conditions (Supplementary Fig. 5B). Given the upregulation of *ZEB1* mRNA, H1650 shSCR and shB56α cells were transduced with *ZEB1* shRNA to generate stable ZEB1 knockdown cells (shSCR shZEB1, shB56α1 shZEB1, shB56α2 shZEB1) to determine the contribution of ZEB1 to the EMT phenotype. Knockdown of ZEB1 did not lead to a significant restoration of E-cadherin or a decrease in Vimentin expression by either protein or mRNA expression (Supplementary Fig. 6A–F). However, consistent with previous reports [33, 34], shZEB1 resulted in a decrease in cell viability over the course of 96 h post-transduction (Supplementary Fig. 6G). These results suggest that ZEB1 contributes to the transcriptional rewiring of NSCLC cells in response to PP2A-B56α suppression and is necessary to maintain the survival of shB56α cells in an EMT state, but is not sufficient to restore epithelial cell markers. Using the Metascape Molecular Complex Detection (MCODE) [35] algorithm to identify significant direct protein-protein interaction networks, we determined that cell-cell adhesion, formation of the cornified envelope, and epithelial cell differentiation networks were significantly enriched in the downregulated proteins (Fig. 5C and Supplementary Data Table 3). Within individual MCODE clusters (MCODE 1-7), there was significant enrichment in desmosome, actin, intermediate filament, and E-Cadherin organization (Supplementary Fig. 7A and Supplementary Data Table 3). These findings, taken together with the fact that the majority of significantly altered proteins were downregulated in shB56α cells (785 versus 657), suggest that PP2A-B56α may function to maintain NSCLC epithelial differentiation programs in order to suppress cellular plasticity.

Within the significantly upregulated proteins in shB56α cells, there was a strong enrichment for signaling programs regulating cell cycle, as well as chromatin organization and mRNA processing (Fig. 5D and Supplementary Data Table 4). These enrichments were consistent with the increased proliferative capacity of shB56α cells (Supplementary Fig. 3D) and support a potential role for B56α in regulating large-scale gene expression programs. Indeed, similar enrichments have been previously identified in response to either a loss of the PP2A catalytic or A subunits [36]. Analysis of transcription factor target interactions identified an enrichment of genes regulated by transcription factors implicated in oncogenesis and EMT, including SP1, FOSL2, KLF8, and MYC (Fig. 5E and Supplementary Data Table 5) [37–45]. Additionally, there was a strong MYC-centered network found within the top 15% of upregulated proteins that was significantly enriched for factors that regulate EMT signaling programs, including Transforming growth factor beta 1 (TGFB1), Tissue Inhibitor of Metalloproteinases 1 (TIMP1), N-cadherin (CDH2), and High Mobility Group AT-Hook 2 (HMGA2) (Fig. 5F and Supplementary Fig. 7B). Of particular interest, this node included AXL, a tyrosine kinase receptor and known driver of EMT and acquired resistance to EGFR targeted therapies in NSCLC [4, 46]. Furthermore, of the kinases implicated in EGFR-mutant NSCLC tumor progression, AXL was the only factor upregulated with B56α suppression (Fig. 5G) and is known to increase MYC activity to exacerbate oncogenesis, potentially implicating a broad regulation of these convergent pathways [4, 46, 47]. Finally, there were 71 significantly altered proteins involved in the epigenetic regulation of gene transcription (GO: 0040029). Specifically, six proteins within the chromatin remodeling NuRD (Nucleosome Remodeling and Deacetylation) complex were significantly increased at the total protein level (Supplementary Fig. 8A and Supplementary Data Table 6). These findings are consistent with studies supporting a role for PP2A in epigenetics [36, 48] and suggest these mechanisms likely contribute in part to the large-scale protein expression changes seen in shB56α cells.

Consistent with the phosphatase function of PP2A-B56α, there were 1129 phospho-sites found to be significantly increased in shB56α cells, compared to 499 that were significantly downregulated (Log_2_ > 1, *p* < 0.05) (Fig. 5H and Supplementary Data Table 7). The unique proteins that displayed increased phosphorylation in shB56α cells were enriched for cell cycle, RNA splicing and processing, and transcriptional regulation pathways, as well as MYC-regulated target genes (Fig. 5I, J and Supplementary Data Table 8 and 9). As a validation of our system, we were able to identify at least 20 phospho-sites implicated to be regulated by PP2A (Supplementary Data Table 10). Furthermore, of the top 100 upregulated phospho-proteins, 70% contained a predicted PP2A-B56-specific short linear binding motifs (SLiMs) [49], indicating that both direct and indirect PP2A targets are altered in shB56α cells. Within the top 100 differential phosphorylated proteins, several factors that are strongly implicated in NSCLC metastasis or EMT had upregulated phospho-sites near B56 SLiM motifs, including Fos-like antigen 1 (FOSL1), A-kinase anchoring protein 12 (AKAP12), Vimentin, and HDAC2 (Histone deacetylase 2) (Supplementary Fig. 8B) [50–52]. Of particular interest, HDAC2, a component of the NuRD complex, has been implicated in the negative regulation of E-cadherin and is a known PP2A target [36]. This factor displayed a significant increase in phosphorylation at S394 in shB56α cells, a site that is critical for HDAC2 activity and resides proximal to a predicted PP2A B56 SLiM (Supplementary Fig. 8B) [36]. Combined, the changes in the H1650 phosphoproteome suggest that suppression of B56α dynamically rewires cells, potentially alleviating the tight regulation of pathways necessary to maintain cellular homeostasis and, thereby, reducing the threshold for cellular plasticity.

### Suppression of PP2A-B56α enhances the metastatic potential of NSCLC in vivo

**Fig. 6:**
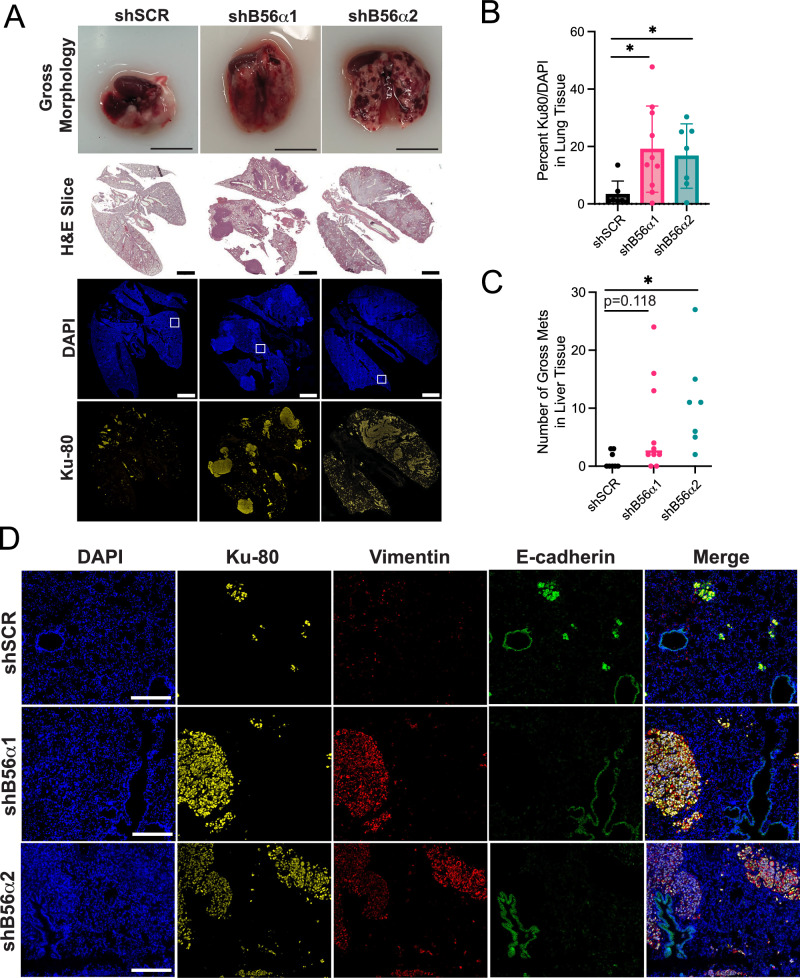
B56α negatively regulates NSCLC metastatic potential in vivo. **A** Representative gross morphology images (scale bar = 1 cm), H&E slices (scale bar = 2 mm), and immunofluorescence for DAPI and Ku-80 (scale bar = 2 mm) in lungs harvested from 25 mice 6 weeks post-tail vein injection with either shB56α or shSCR cells. **B** Quantification of the percent of Ku-80 positive nuclei in the lung from shB56α and shSCR injected mice at endpoint. **C** Quantification of the number of gross liver tumor nodules in each condition. For **B**, **C** Brown-Forsythe and Welch ANOVA, *p < 0.05. **D** Representative immunofluorescence images of DAPI, Ku-80, Vimentin, and E-cadherin taken from the regions denoted panel A (white boxes) (scale bar = 250 μm).

EMT has been associated with increased metastatic capability in a wide variety of tumor types [53]. Our findings suggest that suppression of PP2A-B56α results in EMT in vitro. Therefore, to determine the impact of B56α signaling on metastasis in vivo, 2 million H1650 shSCR and shB56α cells were injected intravenously into 25 immune-compromised mice and tumors were allowed to develop over six weeks, at which point lung and liver tissues were harvested for immunohistological analysis. The majority of shB56α lungs had large, macroscopic tumor nodules and/or widespread tumor dissemination compared to shSCR as seen by both gross morphology and histology (H&E) (Fig. 6A). To quantify lung colonization, immunofluorescence for the human marker ATP-dependent DNA helicase II subunit 2 (Ku-80) was performed and quantified using QuPath [54]. There was a significant ~5-fold increase in the percent of Ku-80+ tumor cells within shB56α lungs compared to shSCR controls (Fig. 6A, B). In addition to lung tumors, almost all mice injected with shB56α cells formed large macroscopic liver tumors compared to mice injected with shSCR cells, which had almost no liver outgrowth (Fig. 6C and Supplementary Fig. 9A). Furthermore, in many cases the shB56α tumor tissue was so extensive that almost no normal liver tissue remained (Supplementary Fig. 9B). Similar to our in vitro results, the Ku-80+ shSCR tumor cells that were able to colonize the lung remained small clusters that were predominantly E-cadherin positive and Vimentin negative (Fig. 6D). In contrast, shB56α tumors remained E-cadherin negative and Vimentin positive, indicative of an aggressive EMT-like state. These results support our hypothesis that PP2A-B56α functions to suppress NSCLC cellular plasticity and suggest that inhibition of B56α may contribute to both EMT and metastatic potential.

## Discussion

PP2A functions as a key gatekeeper to phosphorylation cascades and provides a key “off” switch to a large number of pathways implicated in cancer. However, our understanding of how PP2A dysregulation impacts NSCLC cellular plasticity and invasion is poorly understood. We have determined that suppression of the specific PP2A subunit, B56α, allows NSCLC cells to lose their epithelial characteristics and gain mesenchymal characteristics, indicative of an EMT. Furthermore, identified changes in EMT markers were accompanied by a significant increase in migratory and invasive capacity as evidenced by 2D migration and 3D spheroid invasion assays in vitro, and tail vein invasion assay in vivo. These invasive phenotypes were associated with a vast number of significant proteomic and phosphoproteomic changes within proteins implicated in invasion, EMT, proliferation, and transcriptional regulation. Together, these findings indicate that PP2A-B56α functions as a key modulator of cell state in NSCLC and that suppression of this subunit leads to drastic cellular reprogramming that leads to aggressive disease.

B56α is one of many PP2A B subunits (4 families, 16 subunits total in addition to splice variants). While some functional redundancy has been observed, studies suggest that the role of individual B subunits in disease states is highly context specific [55]. Here, we demonstrate that suppression of B56α leads to drastic morphological and molecular changes in the EGFR-mutant NSCLC cell lines tested, and that the overexpression of B56α increases epithelial markers and suppresses invasive phenotypes. Conversely, the suppression of B55α was unable to recapitulate these findings. These results indicate that the regulation of NSCLC EMT phenotypes is likely to be B56α-specific. However, we cannot rule out the possibility that suppression of B56α alters the dynamics of the PP2A holoenzyme composition, allowing for other subunits to partially contribute to the observed phenotypes. The spatial and temporal relationships that occur between PP2A subunits are both highly biologically relevant and technically challenging to answer; therefore, this area of research remains an open question within the PP2A field. Given that a large percentage of the top differentially regulated proteins contain B56-specific SLiM domains, it is also possible that many of these targets are co-regulated by multiple PP2A B56 subunits. There are limited examples of such regulation; for example, B55α and B56α display opposing roles in the regulation of MYC at two different phospho-sites in breast cancer [56]. However, since the function of individual PP2A B subunits is highly dependent on cellular context, further studies are necessary to identify direct B56α targets versus PP2A as a whole. Finally, as small molecule activators of PP2A (SMAPs) are currently being tested in preclinical studies [16, 26, 55], our findings suggest that pharmacological activation of PP2A may be efficacious in metastatic EGFR-driven NSCLC. In fact, previous studies have demonstrated that SMAPs reduce subcutaneous xenograft tumor growth in EGFR tyrosine kinase inhibitors (TKI) resistant NSCLC [57].

Through our phospho-proteomics analysis, we identified a significant downregulation of proteins involved in cell-cell adhesion and an upregulation of factors involved in EMT, including both MYC and MYC-regulated pathways. B56α is known to dephosphorylate MYC at S62, resulting in MYC’s proteasomal degradation [16, 25, 26, 28]. Therefore, enrichment of MYC signaling pathways highlights the strong reliance of EGFR-mutant NSCLC on the posttranslational regulation of this transcription factor by PP2A and implicates disruption of the PP2A-MYC axis as a potential driver of EMT. In addition to MYC, there were a wide variety of factors implicated in the mesenchymal reprogramming of cancer cells, including HMGA1/2, FOSL1, and TGFβ. As a whole, EMT is a process driven by transcriptional reprogramming in response to increased expression and/or activation of classical EMT-TF such as ZEB1, SNAI1, and TWIST1. While H1650 shB56α cells are reliant on ZEB1 for survival, the knockdown of ZEB1 does not result in a reversal of the EMT phenotype. These findings are consistent with previous studies indicating that mesenchymal to epithelial transition (MET) is not just the reverse of EMT, but rather is mediated by unique factors/events [53, 58]. For instance, ZEB1 is known to associate with HDAC2 to repress *CDH1* gene expression [59, 60]. In shB56α cells, expression of both ZEB1 and HDAC2 is significantly increased, including key phosphorylation sites implicated in activity. However, the loss of ZEB1 does not restore E-cadherin expression, suggesting that other epigenetic and transcriptional events need to occur to initiate MET in these cells. Given that a large number of proteins involved in epigenetics are altered with suppression of B56α, it is possible that epithelial genes are co-regulated or that functionally redundant mechanisms negatively regulate these factors. Taken together with the loss of epithelial markers, we hypothesize that the B56α functions to restrict posttranslational signaling in order to maintain epithelial cell states. The suppression of this subunit potentially alleviates this restrictive programming, poising cells for EMT.

Therapeutic strategies targeting the EGFR pathway have provided immense clinical benefit for patients with activating genomic alterations in EGFR, with EGFR TKIs such as Osimertinib increasing progression-free survival from 10.2 months to 18.9 months and overall survival from 31.8 months to 38.6 months when compared to first-line platinum-based therapies [61, 62]. However, acquired drug resistance continues to be a clinical challenge. EMT drives therapeutic resistance in a broad range of tumors and therapeutics, including in EGFR-mutant NSCLCs relapsing on Osimertinib and earlier generation EGFR inhibitors [63, 64]. Suppression of the B56α subunit of PP2A leads to a near-complete loss of pY1068 EGFR, suggesting that shB56α cells function independently of EGFR signaling despite mutant EGFR being the driving mutation in these cells. This finding is consistent with previous reports demonstrating that loss of EGFR signaling can occur during EMT [65]. EGFR Y1068 is not a direct target of PP2A. Therefore, these results suggest that knockdown of B56α potentially alters the expression of EGFR binding partners critical for maintaining pY1068 levels. This regulation may be epigenetic in nature, as phosphorylation at this site is only partially rescued after prolonged exposure to exogenous B56α. While our studies suggest that shB56α results in a loss of pY1068 EGFR and reduced Osimertinib sensitivity, future studies will aim to interrogate the dependency of PP2A-regulated EMT on therapeutic response, and whether PP2A perturbation in primary patient tumors (e.g., high CIP2A (PP2A inhibitor)) alters therapeutic response.

In summary, these studies address a critical gap in our understanding of the posttranslational mechanisms that govern NSCLC EMT. Our findings strongly implicate PP2A-B56α as a critical regulator of cellular plasticity in EGFR mutant NSCLC and highlight the need for continued studies interrogating the therapeutic potential of PP2A in the metastatic setting.

## Supplementary information

PDF_Supplementary Figures_merge

SupplementaryDataTables

## Data Availability

All data analyzed during this study are included in this published article and associated supplementary information files. Data and cell lines generated during the current study are available from the corresponding author on reasonable request or a material transfer agreement.

## References

[1] Murphy PK, Sellers ME, Bonds SH, Scott S. The SEER Program’s longstanding commitment to making cancer resources available. JNCI Monogr. 2024;2024:118–22. 10.1093/jncimonographs/lgae028 PMC11300012 39102882

[2] Chen Y, Deng J, Liu Y, Wang H, Zhao S, He Y, et al. Analysis of metastases in non-small cell lung cancer patients with epidermal growth factor receptor mutation. Ann Transl Med. 2021;9:206. 33708833 10.21037/atm-20-2925 PMC7940889

[3] Huang Y, Hong W, Wei X. The molecular mechanisms and therapeutic strategies of EMT in tumor progression and metastasis. J Hematol Oncol. 2022;15:129. 36076302 10.1186/s13045-022-01347-8 PMC9461252

[4] Leonetti A, Sharma S, Minari R, Perego P, Giovannetti E, Tiseo M. Resistance mechanisms to osimertinib in EGFR-mutated non-small cell lung cancer. Br J Cancer. 2019;121:725–37. 31564718 10.1038/s41416-019-0573-8 PMC6889286

[5] Dudás J, Ladányi A, Ingruber J, Steinbichler TB, Riechelmann H. Epithelial to mesenchymal transition: a mechanism that fuels cancer radio/chemoresistance. Cells. 2020;9:428. 32059478 10.3390/cells9020428 PMC7072371

[6] Saitoh M. Transcriptional regulation of EMT transcription factors in cancer. Semin Cancer Biol. 2023;97:21–29. 37802266 10.1016/j.semcancer.2023.10.001

[7] Yu X, He T, Tong Z, Liao L, Huang S, Fakhouri WD, et al. Molecular mechanisms of TWIST1-regulated transcription in EMT and cancer metastasis. EMBO Rep. 2023;24:e56902. 37680145 10.15252/embr.202356902 PMC10626429

[8] Nam M-W, Kim C-W, Choi K-C. Epithelial-mesenchymal transition-inducing factors involved in the progression of lung cancers. Biomol Ther. 2022;30:213–20. 10.4062/biomolther.2021.178 PMC9047489 35039464

[9] Pallier K, Cessot A, Côté JF, Just PA, Cazes A, Fabre E, et al. TWIST1, a new determinant of epithelial to mesenchymal transition in EGFR-mutated lung adenocarcinoma. PLoS ONE. 2012;7:e29954. 22272264 10.1371/journal.pone.0029954 PMC3260187

[10] Kang E, Seo J, Yoon H, Cho S. The post-translational regulation of epithelial–mesenchymal transition-inducing transcription factors in cancer metastasis. Int J Mol Sci. 2021;22:3591. 33808323 10.3390/ijms22073591 PMC8037257

[11] Ruvolo PP. The broken “Off” switch in cancer signaling: PP2A as a regulator of tumorigenesis, drug resistance, and immune surveillance. BBA Clin. 2016;6:87–99. 27556014 10.1016/j.bbacli.2016.08.002 PMC4986044

[12] Schuhmacher D, Sontag J-M, Sontag E. Protein phosphatase 2A: more than a passenger in the regulation of epithelial cell–cell junctions. Front Cell Dev Biol. 2019;7:30. 30895176 10.3389/fcell.2019.00030 PMC6414416

[13] Sablina AA, Hector M, Colpaert N, Hahn WC. Identification of PP2A complexes and pathways involved in cell transformation. Cancer Res. 2010;70:10474–84. 21159657 10.1158/0008-5472.CAN-10-2855 PMC3056544

[14] Janghorban M, Langer EM, Wang X, Zachman D, Daniel CJ, Hooper J, et al. The tumor suppressor phosphatase PP2A-B56α regulates stemness and promotes the initiation of malignancies in a novel murine model. PLoS ONE. 2017;12:e0188910. 29190822 10.1371/journal.pone.0188910 PMC5708644

[15] Mannava S, Omilian AR, Wawrzyniak JA, Fink EE, Zhuang D, Miecznikowski JC, et al. PP2A-B56α controls oncogene-induced senescence in normal and tumor human melanocytic cells. Oncogene. 2012;31:1484–92. 21822300 10.1038/onc.2011.339 PMC3213274

[16] Tinsley SL, Chianis E, Shelley RA, Mall GK, Dhiman A, Baral G, et al. KRAS-mediated upregulation of CIP2A promotes suppression of PP2A-B56α to initiate pancreatic cancer development. Oncogene. 2024;43:3673–87. 39443726 10.1038/s41388-024-03196-w PMC12573297

[17] Raoof S, Mulford IJ, Frisco-Cabanos H, Nangia V, Timonina D, Labrot E, et al. Targeting FGFR overcomes EMT-mediated resistance in EGFR mutant non-small cell lung cancer. Oncogene. 2019;38:6399–413. 31324888 10.1038/s41388-019-0887-2 PMC6742540

[18] Crystal AS, Shaw AT, Sequist LV, Friboulet L, Niederst MJ, Lockerman EL, et al. Patient-derived models of acquired resistance can identify effective drug combinations for cancer. Science. 2014;346:1480–6. 25394791 10.1126/science.1254721 PMC4388482

[19] Benton A, Moriarty NM, Terwilliger E, Liu B, Murphy A, Maluvac H, et al. miR-497 target gene regulatory network in angiosarcoma. Mol Cancer Res. 2024;22:879–90. 38771248 10.1158/1541-7786.MCR-23-1075 PMC11374500

[20] Grecco GG, Huang JY, Muñoz B, Doud EH, Hines CD, Gao Y, et al. Sex-dependent synaptic remodeling of the somatosensory cortex in mice with prenatal methadone exposure. Adv Drug Alcohol Res. 2022;2:10400. 37829495 10.3389/adar.2022.10400 PMC10569410

[21] McCracken NA, Liu H, Runnebohm AM, Wijeratne H, Wijeratne AB, Staschke KA, et al. Obtaining functional proteomics insights from thermal proteome profiling through optimized melt shift calculation and statistical analysis with InflectSSP. Mol Cell Proteom: MCP. 2023;22:100630. 37562535 10.1016/j.mcpro.2023.100630 PMC10494267

[22] Orsburn BC. Proteome Discoverer—a community-enhanced data processing suite for protein informatics. Proteomes. 2021;9:15. 33806881 10.3390/proteomes9010015 PMC8006021

[23] Zhou Y, Zhou B, Pache L, Chang M, Khodabakhshi AH, Tanaseichuk O, et al. Metascape provides a biologist-oriented resource for the analysis of systems-level datasets. Nat Commun. 2019;10:1523. 30944313 10.1038/s41467-019-09234-6 PMC6447622

[24] Győrffy B. Transcriptome-level discovery of survival-associated biomarkers and therapy targets in non-small-cell lung cancer. Br J Pharmacol. 2024;181:362–74. 37783508 10.1111/bph.16257

[25] Arnold HK, Sears RC. A tumor suppressor role for PP2A-B56α through negative regulation of c-Myc and other key oncoproteins. Cancer Metastasis Rev. 2008;27:147–58. 18246411 10.1007/s10555-008-9128-9 PMC3045695

[26] Allen-Petersen BL, Risom T, Feng Z, Wang Z, Jenny ZP, Thoma MC, et al. Activation of PP2A and inhibition of mTOR synergistically reduce MYC signaling and decrease tumor growth in pancreatic ductal adenocarcinoma. Cancer Res. 2018;79:209–19. 30389701 10.1158/0008-5472.CAN-18-0717 PMC6318036

[27] Farrington CC, Yuan E, Mazhar S, Izadmehr S, Hurst L, Allen-Petersen BL, et al. Protein phosphatase 2A activation as a therapeutic strategy for managing MYC-driven cancers. J Biol Chem. 2019;295:757–70. 31822503 10.1074/jbc.RA119.011443 PMC6970930

[28] Arnold HK, Sears RC. Protein phosphatase 2A regulatory subunit B56α associates with c-Myc and negatively regulates c-Myc accumulation. Mol Cell Biol. 2006;26:2832–44. 16537924 10.1128/MCB.26.7.2832-2844.2006 PMC1430332

[29] Tsherniak A, Vazquez F, Montgomery PG, Weir BA, Kryukov G, Cowley GS, et al. Defining a cancer dependency map. Cell. 2017;170:564–76.e16. 28753430 10.1016/j.cell.2017.06.010 PMC5667678

[30] Wang C-Y, Chao TT, Chang FY, Chen YL, Tsai YT, Lin HI, et al. CIP2A mediates erlotinib-induced apoptosis in non-small cell lung cancer cells without EGFR mutation. Lung Cancer. 2014;85:152–60. 24954871 10.1016/j.lungcan.2014.05.024

[31] Khanna A, Okkeri J, Bilgen T, Tiirikka T, Vihinen M, Visakorpi T, et al. ETS1 mediates MEK1/2-dependent overexpression of cancerous inhibitor of protein phosphatase 2A (CIP2A) in human cancer cells. PLoS ONE. 2011;6:e17979. 21445343 10.1371/journal.pone.0017979 PMC3062549

[32] Han H, Cho JW, Lee S, Yun A, Kim H, Bae D, et al. TRRUST v2: an expanded reference database of human and mouse transcriptional regulatory interactions. Nucleic Acids Res. 2018;46:D380–6. 29087512 10.1093/nar/gkx1013 PMC5753191

[33] Sánchez-Tilló E, Pedrosa L, Vila I, Chen Y, Győrffy B, Sánchez-Moral L, et al. The EMT factor ZEB1 paradoxically inhibits EMT in BRAF-mutant carcinomas. JCI Insight. 2023;8:e164629. 37870961 10.1172/jci.insight.164629 PMC10619495

[34] Schuhwerk H, Kleemann J, Gupta P, van Roey R, Armstark I, Kreileder M, et al. The EMT transcription factor ZEB1 governs a fitness-promoting but vulnerable DNA replication stress response. Cell Rep. 2022;41:111819. 36516781 10.1016/j.celrep.2022.111819

[35] Bader GD, Hogue CW. An automated method for finding molecular complexes in large protein interaction networks. BMC Bioinform. 2003;4:2. 10.1186/1471-2105-4-2 PMC149346 12525261

[36] Aakula A, Sharma M, Tabaro F, Nätkin R, Kamila J, Honkanen H, et al. RAS and PP2A activities converge on epigenetic gene regulation. Life Sci Alliance. 2023;6:e202301928. 36858798 10.26508/lsa.202301928 PMC9979842

[37] Kim IK, Lee YS, Kim HS, Dong SM, Park JS, Yoon DS. Specific protein 1(SP1) regulates the epithelial-mesenchymal transition via lysyl oxidase-like 2(LOXL2) in pancreatic ductal adenocarcinoma. Sci Rep. 2019;9:5933. 30976063 10.1038/s41598-019-42501-6 PMC6459819

[38] Zhang H, Zhang G, Zhang J, Xiao M, Cui S, Wu S, et al. Transcription factor SP1 and oncoprotein PPP1R13L regulate nicotine-induced epithelial-mesenchymal transition in lung adenocarcinoma via a feedback loop. Biochem Pharmacol. 2022;206:115344. 36372331 10.1016/j.bcp.2022.115344

[39] Yin J, Hu W, Fu W, Dai L, Jiang Z, Zhong S, et al. HGF/MET-regulated epithelial-mesenchymal transitions and metastasis by FOSL2 in non-small cell lung cancer. OncoTargets Ther. 2019;12:9227–37. 10.2147/OTT.S217595 PMC6842307 31807006

[40] Wang X, Lu H, Urvalek AM, Li T, Yu L, Lamar J, et al. KLF8 promotes human breast cancer cell invasion and metastasis by transcriptional activation of MMP9. Oncogene. 2011;30:1901–11. 21151179 10.1038/onc.2010.563 PMC3952074

[41] Jing P, Xie N, Zhao N, Zhu X, Li P, Gao G, et al. miR-24-3p/KLF8 signaling axis contributes to LUAD metastasis by regulating EMT. J Immunol Res. 2020;2020:4036047. 32411796 10.1155/2020/4036047 PMC7204180

[42] Wang X, Zheng M, Liu G, Xia W, McKeown-Longo PJ, Hung MC, et al. Krüppel-Like factor 8 induces epithelial to mesenchymal transition and epithelial cell invasion. Cancer Res. 2007;67:7184–93. 17671186 10.1158/0008-5472.CAN-06-4729

[43] Meškytė EM, Keskas S, Ciribilli Y. MYC as a multifaceted regulator of tumor microenvironment leading to metastasis. Int J Mol Sci. 2020;21:7710. 33081056 10.3390/ijms21207710 PMC7589112

[44] Yin S, Cheryan VT, Xu L, Rishi AK, Reddy KB. Myc mediates cancer stem-like cells and EMT changes in triple-negative breast cancer cells. PLoS ONE. 2017;12:e0183578. 28817737 10.1371/journal.pone.0183578 PMC5560738

[45] Cho KB, Cho MK, Lee WY, Kang KW. Overexpression of c-myc induces epithelial-mesenchymal transition in mammary epithelial cells. Cancer Lett. 2010;293:230–9. 20144848 10.1016/j.canlet.2010.01.013

[46] Taniguchi H, Yamada T, Wang R, Tanimura K, Adachi Y, Nishiyama A, et al. AXL confers intrinsic resistance to osimertinib and advances the emergence of tolerant cells. Nat Commun. 2019;10:259. 30651547 10.1038/s41467-018-08074-0 PMC6335418

[47] Sun X, Chen H, You S, Tian Z, Wang Z, Liu F, et al. AXL upregulates c-Myc expression through AKT and ERK signaling pathways in breast cancers. Mol Clin Oncol. 2023;18:22. 36844467 10.3892/mco.2023.2618 PMC9944620

[48] Tinsley SL, Allen-Petersen BL. PP2A and cancer epigenetics: a therapeutic opportunity waiting to happen. Nar Cancer. 2022;4:zcac002. 35118387 10.1093/narcan/zcac002 PMC8807117

[49] Hertz EPT, Kruse T, Davey NE, López-Méndez B, Sigurðsson JO, Montoya G, et al. A conserved motif provides binding specificity to the PP2A-B56 phosphatase. Mol Cell. 2016;63:686–95. 27453045 10.1016/j.molcel.2016.06.024

[50] Casalino L, Talotta F, Matino I, Verde P. FRA-1 as a regulator of EMT and metastasis in breast cancer. Int J Mol Sci. 2023;24:8307. 37176013 10.3390/ijms24098307 PMC10179602

[51] Kim N, Jung J. Exploring the role of AKAP12 in cancer progression and therapeutic implication. Drug Targets Ther. 2025;4:59–66.

[52] Zhao Y, Liu MJ, Zhang L, Yang Q, Sun QH, Guo JR, et al. High mobility group A1 (HMGA1) promotes the tumorigenesis of colorectal cancer by increasing lipid synthesis. Nat Commun. 2024;15:9909. 39548107 10.1038/s41467-024-54400-0 PMC11568219

[53] Jolly MK, Ware KE, Gilja S, Somarelli JA, Levine H. EMT and MET: necessary or permissive for metastasis? Mol Oncol. 2017;11:755–69. 28548345 10.1002/1878-0261.12083 PMC5496498

[54] Bankhead P, Loughrey MB, Fernández JA, Dombrowski Y, McArt DG, Dunne PD, et al. QuPath: open source software for digital pathology image analysis. Sci Rep. 2017;7:16878. 29203879 10.1038/s41598-017-17204-5 PMC5715110

[55] Haanen TJ, O’Connor CM, Narla G. Biased holoenzyme assembly of protein phosphatase 2A (PP2A): From cancer to small molecules. J Biol Chem. 2022;298:102656. 36328247 10.1016/j.jbc.2022.102656 PMC9707111

[56] Zhang L, Zhou H, Li X, Vartuli RL, Rowse M, Xing Y, et al. Eya3 partners with PP2A to induce c-Myc stabilization and tumor progression. Nat Commun. 2018;9:1047. 29535359 10.1038/s41467-018-03327-4 PMC5849647

[57] Tohmé R, Izadmehr S, Gandhe S, Tabaro G, Vallabhaneni S, Thomas A, et al. Direct activation of PP2A for the treatment of tyrosine kinase inhibitor–resistant lung adenocarcinoma. JCI Insight. 2019;4:e125693. 30830869 10.1172/jci.insight.125693 PMC6478418

[58] Kim HY, Jackson TR, Davidson LA. On the role of mechanics in driving mesenchymal-to-epithelial transitions. Semin Cell Dev Biol. 2017;67:113–22. 27208723 10.1016/j.semcdb.2016.05.011 PMC5115991

[59] Aghdassi A, Sendler M, Guenther A, Mayerle J, Behn CO, Heidecke CD, et al. Recruitment of histone deacetylases HDAC1 and HDAC2 by the transcriptional repressor ZEB1 downregulates E-cadherin expression in pancreatic cancer. Gut. 2012;61:439–48. 22147512 10.1136/gutjnl-2011-300060

[60] Manshouri R, Coyaud E, Kundu ST, Peng DH, Stratton SA, Alton K, et al. ZEB1/NuRD complex suppresses TBC1D2b to stimulate E-cadherin internalization and promote metastasis in lung cancer. Nat Commun. 2019;10:5125. 31719531 10.1038/s41467-019-12832-z PMC6851102

[61] Ramalingam SS, Vansteenkiste J, Planchard D, Cho BC, Gray JE, Ohe Y, et al. Overall survival with osimertinib in untreated, EGFR-mutated advanced NSCLC. N Engl J Med. 2019;382:41–50. 31751012 10.1056/NEJMoa1913662

[62] Soria J-C, Ohe Y, Vansteenkiste J, Reungwetwattana T, Chewaskulyong B, Lee KH, et al. Osimertinib in untreated EGFR-mutated advanced non–small-cell lung cancer. N Engl J Med. 2017;378:113–25. 29151359 10.1056/NEJMoa1713137

[63] Sequist LV, Waltman BA, Dias-Santagata D, Digumarthy S, Turke AB, Fidias P, et al. Genotypic and histological evolution of lung cancers acquiring resistance to EGFR inhibitors. Sci Transl Med. 2011;3:75ra26. 21430269 10.1126/scitranslmed.3002003 PMC3132801

[64] Zatzman M, Quintanal-Villalonga A, Salehi S, Ceglia N, Lee JJ, Pupo AN, et al. Complementary modes of resistance to EGFR TKI in lung adenocarcinoma through MAPK activation and cellular plasticity. Preprint at 10.1101/2025.05.07.652714.

[65] Verusingam ND, Chen YC, Lin HF, Liu CY, Lee MC, Lu KH, et al. Generation of osimertinib-resistant cells from epidermal growth factor receptor L858R/T790M mutant non-small cell lung carcinoma cell line. J Chin Méd Assoc. 2020;84:248–54. 10.1097/JCMA.0000000000000438 PMC12966177 33009209

