# Supplementary material for: Suppression of PP2A-B56α drives EMT in EGFR mutant non-small cell lung cancer
Source: Oncogene. 2026 Apr 11;45(20):1861–73. doi: 10.1038/s41388-026-03772-2 (PMC13167467; doi:10.1038/s41388-026-03772-2)
Supplement: Supplementary file 1 — PDF_Supplementary Figures_merge [file 41388_2026_3772_MOESM1_ESM.pdf]

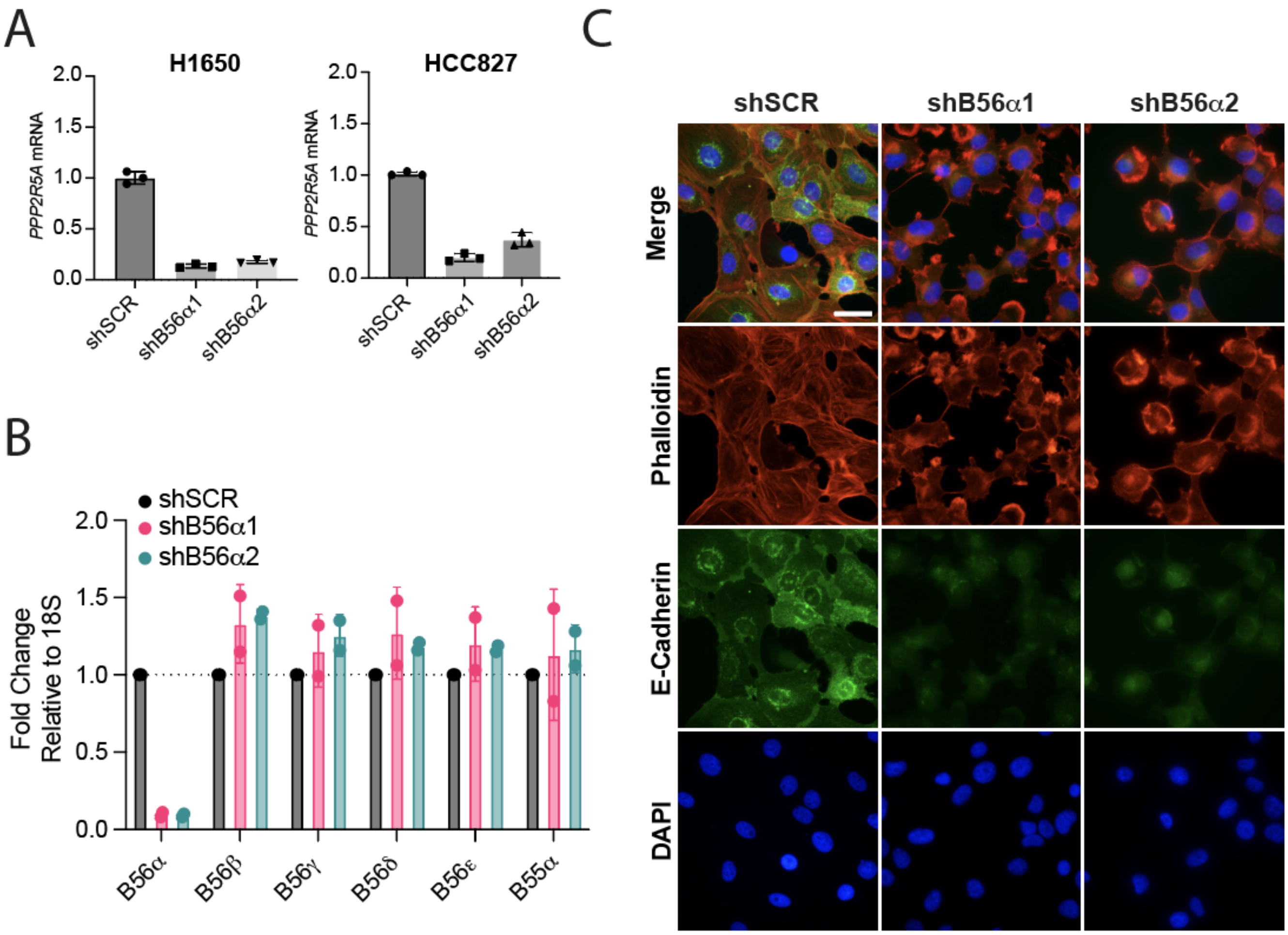

Supplemental Figure 1: A) mRNA expression of B56α (*PPP2R5A*) in H1650 and HCC827 shB56α cells compared to shSCR control. B) mRNA expression of B56 family subunits and B55α in H1650 shB56α compared to shSCR control (n=2 biological replicates). C) Representative images of H1650 cells with immunofluorescence for DAPI (blue), Phalloidin (F-actin, red), and E-cadherin (green)(scale bar = 100μm).

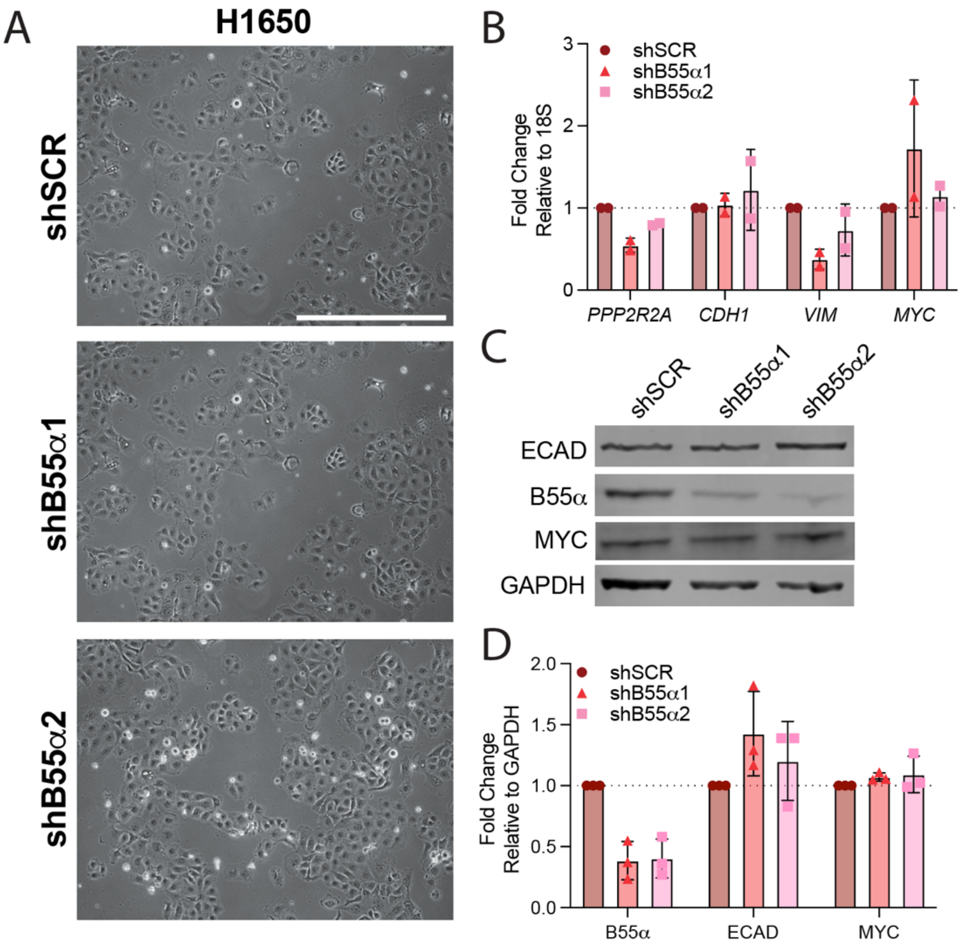

Supplemental Figure 2: A) Morphology of H1650 cells with shRNA mediated stable knockdown of the B55α subunit (shB55α1, shB55α2) compared to shSCR control (scale bar=500μm). B) mRNA expression of B55α (*PPP2R2A*), E-cadherin (*CDH1*), Vimentin (*VIM*) and c-Myc (*MYC*) (n=2 biological replicates). C) Representative western blot of H1650 shB55α cells compared to shSCR control. D) Fold change quantification of C relative to GAPDH (n=3 biological replicates).

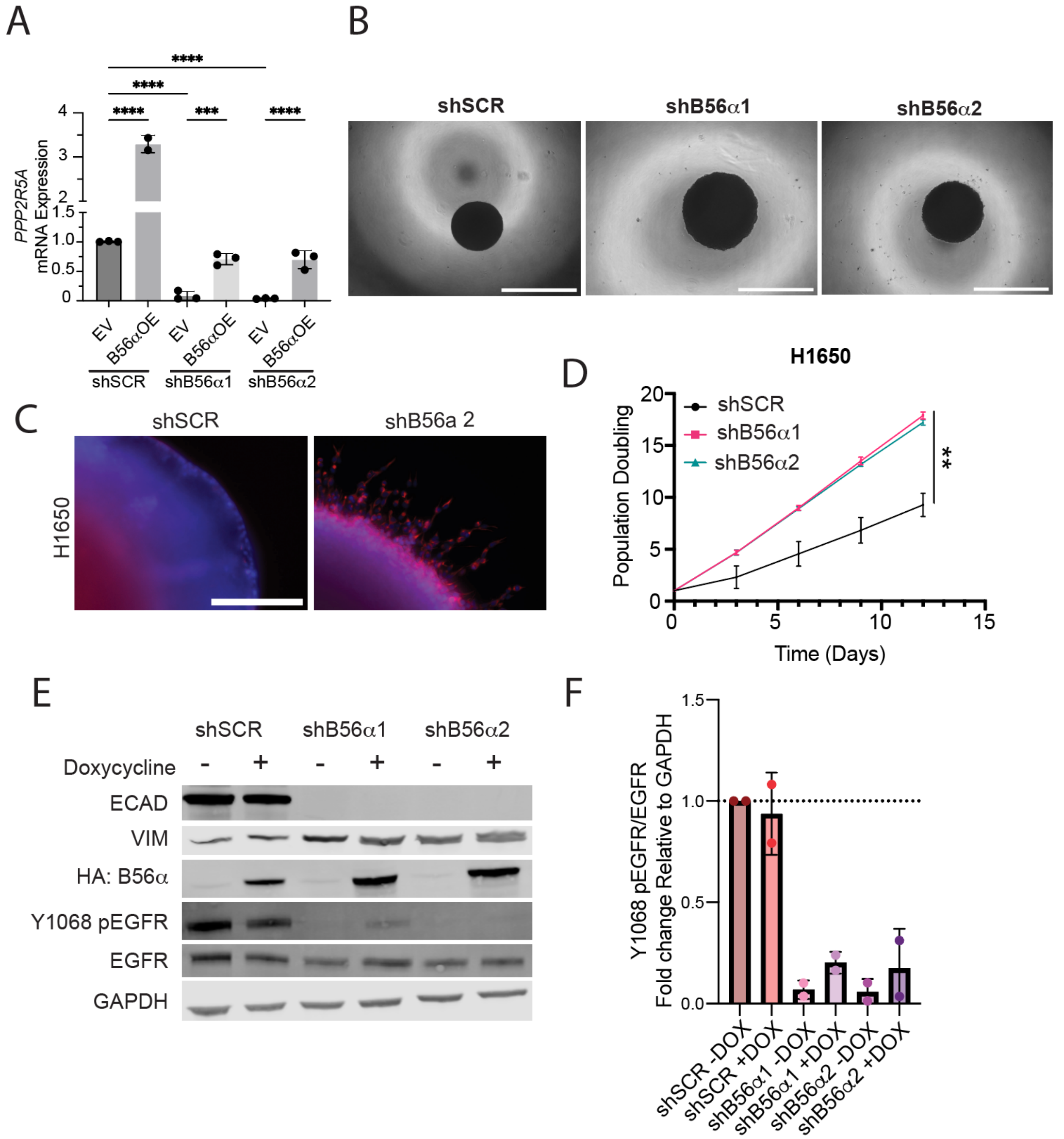

Supplemental Figure 3: A) mRNA expression of B56α (*PPP2R5A*) at the time of plating the transwell migration assay in Figure 3A. B) Representative images of spheroids at the time of embedding the spheroids in Matrigel for the spheroid invasion assay in Figure 3C (scale bar = 1mm). C) Representative immunofluorescent images of DAPI and Phalloidin (F-actin) in H1650 shB56α compared to shSCR (scale bar = 250μm). D) Population doubling of H1650 shB56α cells compared to shSCR control (n=3 biological replicates, ANOVA from area under the curve, $p<0.001$).

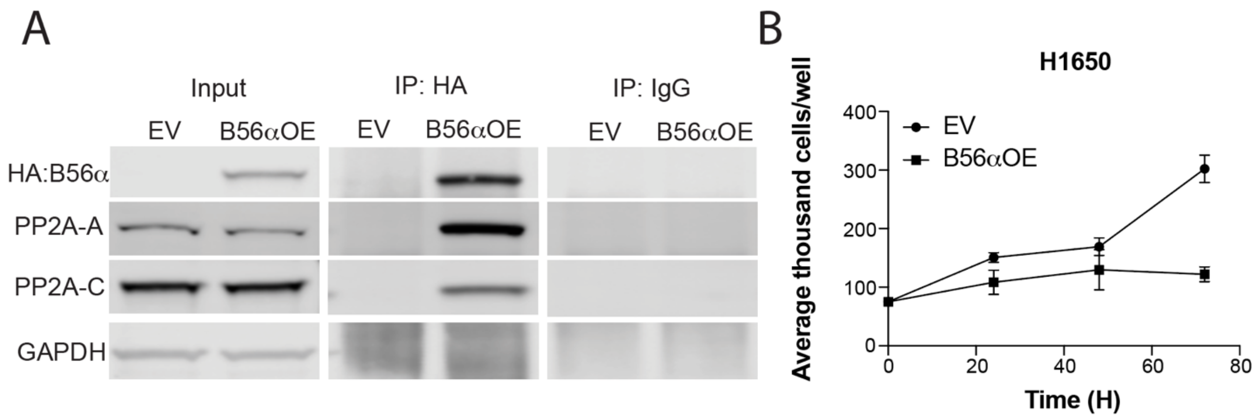

Supplemental Figure 4: A) Immunoprecipitation western blot of HCC827 B56αOE cells compared to EV showing pull down of HA-tagged B56α subunit in association with PP2A-A subunit and PP2A-C subunit (n=1 biological replicate). B) Proliferation of stable overexpression of B56α (B56αOE) in H1650 cell line compared to empty vector control (EV). Shown as 3 technical replicates.

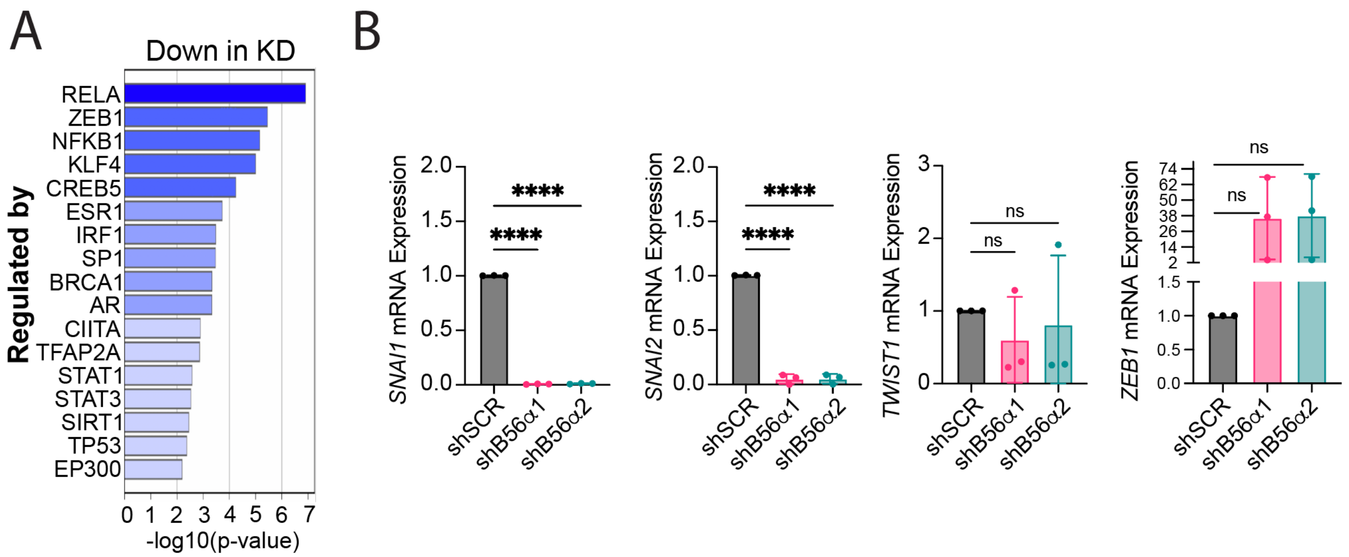

Supplemental Figure 5: A) TRRUST analysis of transcription factor target interactions that are decreased in shB56α total proteomics compared to shSCR control (Log_2FC <-2, $p<0.05$). B) mRNA expression analysis by qRT-PCR of classical EMT-TFs including SNAIL (*SNAI1*), SLUG (*SNAI2*), TWIST (*TWIST1*), and ZEB1 (*ZEB1*) in shB56α compared to shSCR cells (n=3 biological replicates).

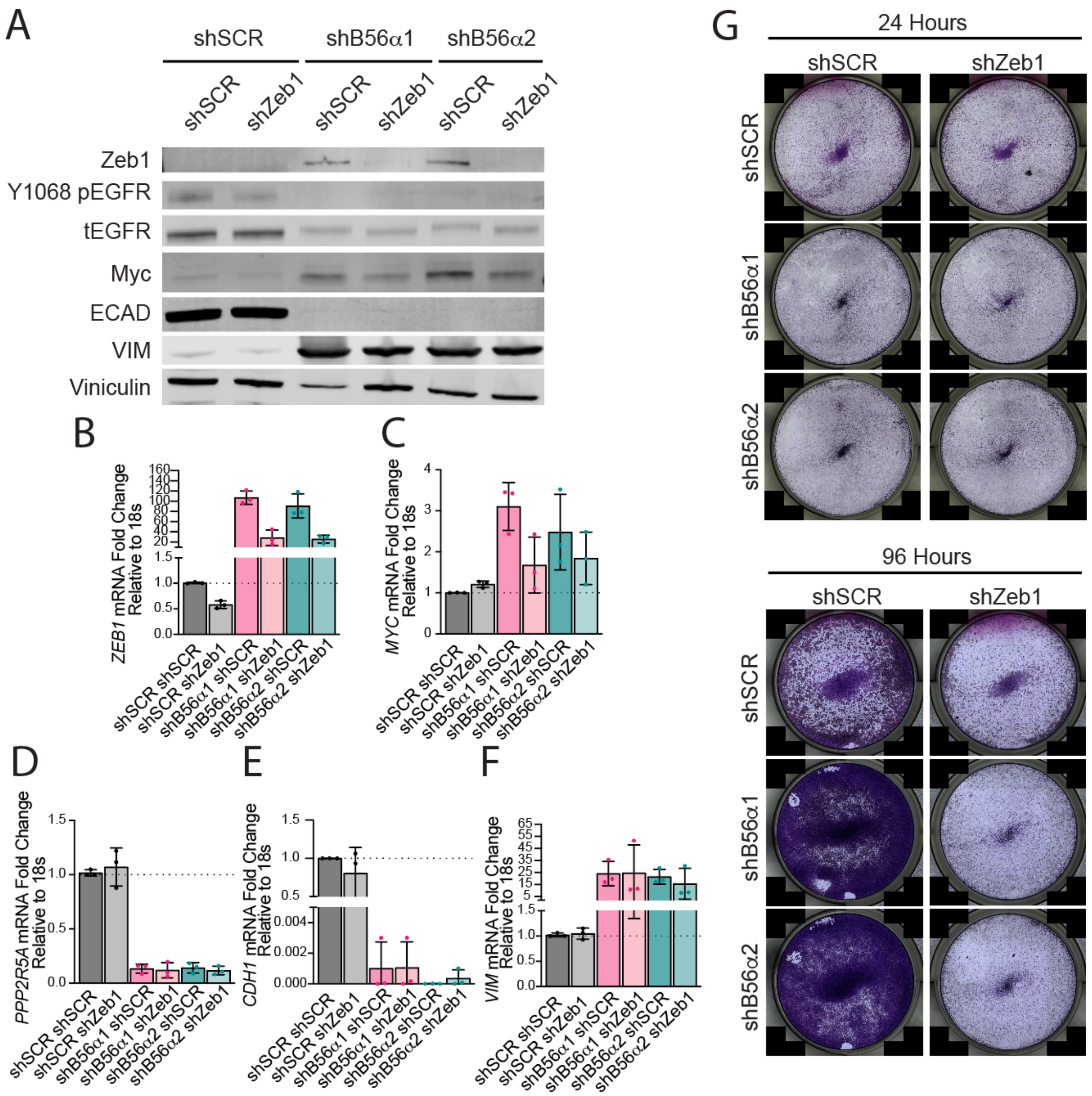

Supplemental Figure 6: A) Representative western blot of H1650 shB56α with knockdown of Zeb1 (shZeb1) for 48 hours. mRNA expression of Zeb1 (B), MYC (C), B56α (D), E-cadherin (E), and Vimentin (F) in H1650 shB56a cells with Zeb1 knockdown compared to shSCR shSCR control (N=3 biological replicates). G) Whole well images of crystal violet fixed and stained H1650 shB56α cells with and without Zeb1 knockdown for 24 hours (top) and 96 hours (bottom).

A

Color	MCODE	GO	Description	Log10(P)
	MCODE_1	GO:0002934	desmosome organization	-18.9
	MCODE_1	R-HSA-6809371	Formation of the cornified envelope	-15.3
	MCODE_1	R-HSA-6805567	Keratinization	-13.6
	MCODE_2	WP383	Striated muscle contraction pathway	-10.5
	MCODE_2	hsa04820	Cytoskeleton in muscle cells	-9.8
	MCODE_2	GO:0007015	actin filament organization	-9.4
	MCODE_3	M184	PID ECADHERIN KERATINOCYTE PATHWAY	-12.1
	MCODE_3	GO:0044331	cell-cell adhesion mediated by cadherin	-11.3
	MCODE_3	M156	PID ECADHERIN NASCENT AJ PATHWAY	-10.9
	MCODE_4	GO:0034340	response to type I interferon	-10.2
	MCODE_4	GO:0051607	defense response to virus	-10.1
	MCODE_4	R-HSA-909733	Interferon alpha/beta signaling	-10.0
	MCODE_5	R-HSA-432720	Lysosome Vesicle Biogenesis	-12.1
	MCODE_5	R-HSA-199992	trans-Golgi Network Vesicle Budding	-10.9
	MCODE_5	R-HSA-432722	Golgi Associated Vesicle Biogenesis	-7.9
	MCODE_6	GO:0045109	intermediate filament organization	-7.2
	MCODE_6	GO:0031424	keratinization	-7.1
	MCODE_6	GO:0045104	intermediate filament cytoskeleton organization	-6.9
	MCODE_7	GO:0045109	intermediate filament organization	-7.8
	MCODE_7	GO:0045104	intermediate filament cytoskeleton organization	-7.5
	MCODE_7	GO:0045103	intermediate filament-based process	-7.5

B

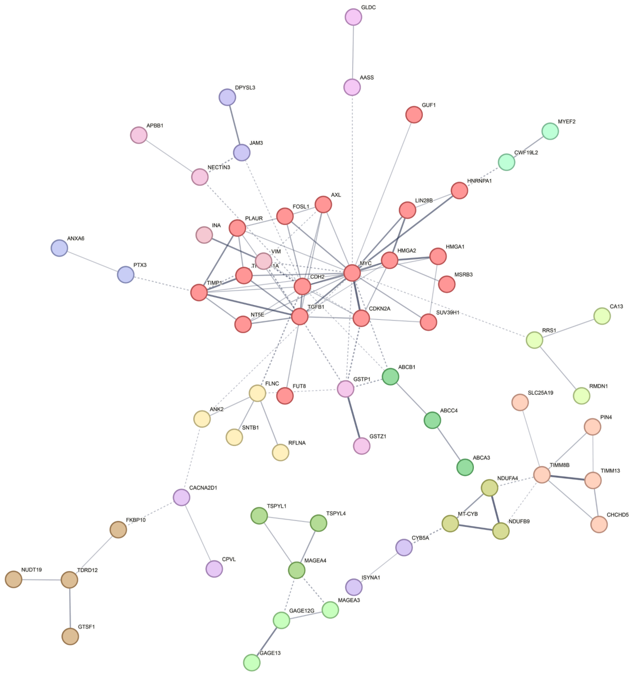

Supplemental Figure 7: A) MCODE clusters from the Metascape MCODE analysis of significantly decreased proteins in the total proteomics (Log_2FC <-2, p<0.05). D) STRING enrichment analysis for top 15% of differentially upregulated proteins in shB56α cells.

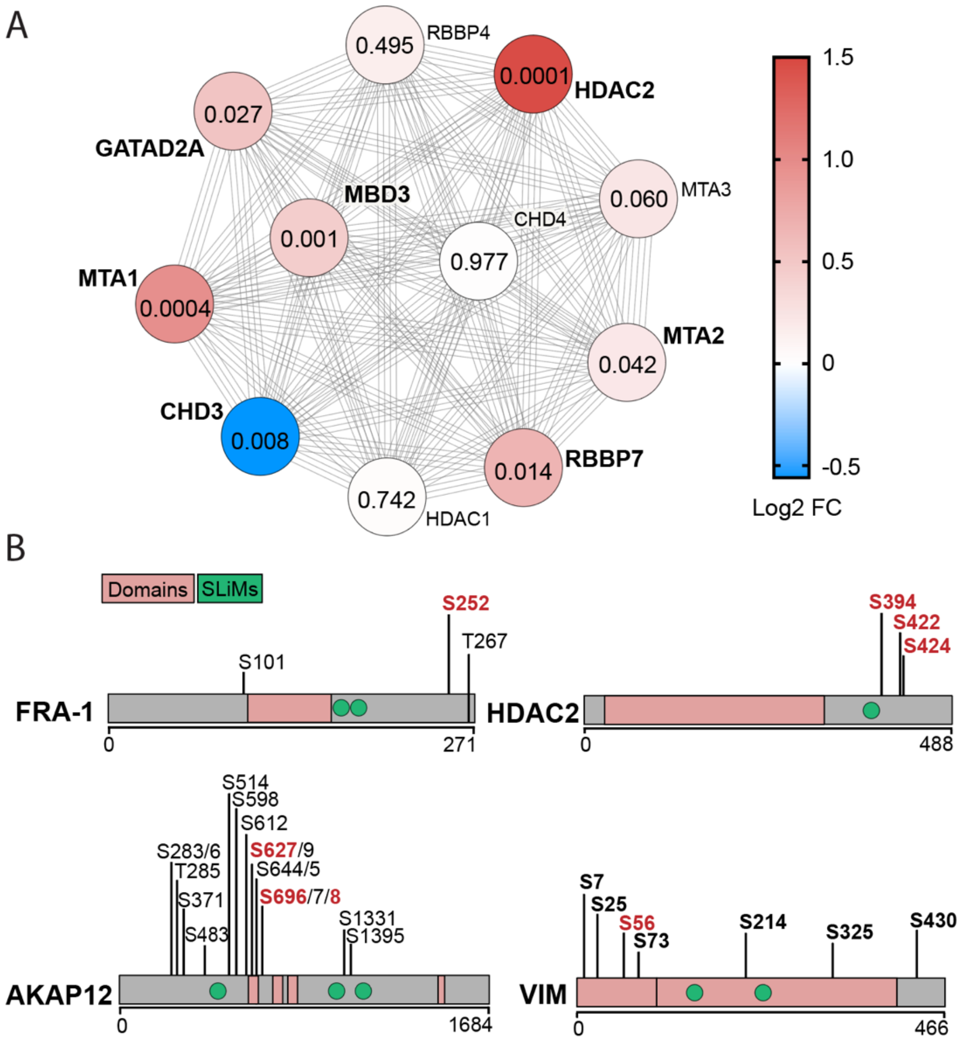

Supplemental Figure 8: A) P-values from total proteomics displayed as a STRING network of NuRD complex proteins. Color coded for Log2 fold change (Log2FC) as shown on the right. Significantly changed proteins are in bold. B) Significantly upregulated phospho-sites on FRA-1, AKAP12, and HDAC2 and VIM in shB56α cells are denoted in black. Red sites indicate known phosphorylation events implicated in EMT or invasion. Major domains (pink). B56 binding motifs, SLiMs (green).

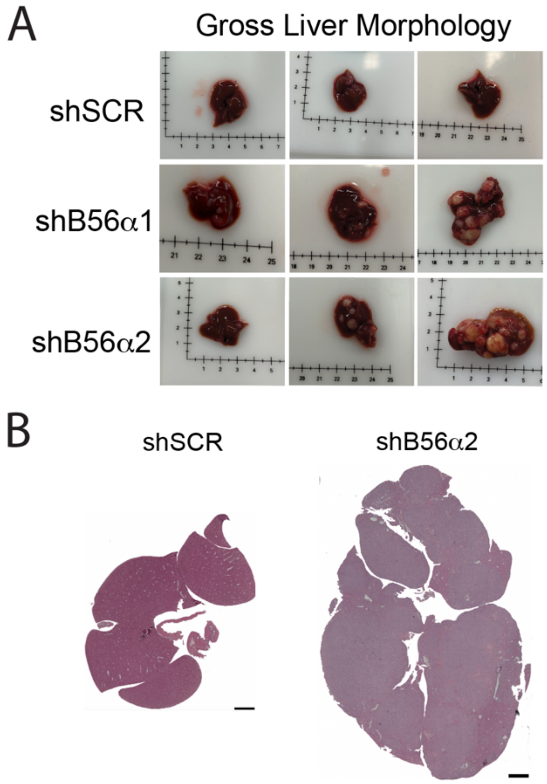

Supplemental Figure 9: A) Gross morphology of liver from mice injected with H1650 shB56α or shSCR control cells representing a low, medium, and high number of metastases for each group. B) H&E of liver showing increased tumor in shB56α2 compared to shSCR (scale bar = 2mm).

Supplemental Methods Table 1: qRT-PCR primer sequences for genes analyzed.

Gene Name	Species	Forward Sequence	Reverse Sequence
***PPP2R5A* B56α**	Human	5'-AgAgCCCTgATTTCCAgCCTA-3'<br>5'-CTTTgCATTgCCACTgAAAg-3'	3'-TTTCCCATAAATTCggTgCAgA-5'<br>3'-CAgCAgTCCTCTgATCAC-5'
***PPP2R2A* B55α**	Human	5'-CATACCAggTgCATgAATACCTC-3'	3'-gggTTATgTCTCgCTTTgTgTTT-5'
CDH1	Human	5'-gAACgCATTgCCACATAC-3'	3'-ACCTTCCATgACAgACCC-5'
VIM	Human	5'-AgTCCACTgAgTACCggAgAC-3'	3'-CATTTCACgCATCTggCgTTC-5'
MYC	Human	5'-CAAACCTCCTCACAgCCCACT-3'	3'-TTCgCCTCTTgACATTCTCCTC-5'
***KIAA1524* (CIP2A)**	Human	5'-gCCACACTgATTCggTgTTTT-3'	3'-TgCCgACAAAgATTTgCCAATA-5'
ZEB1	Human	TTACACCTTTgCATACAgAACCC	TTTACgATTACACCCAgACTgC
TWIST	Human	5'-CCAggTACATCgACTTCCTCTA-3'	3'-CCATCCTCCAgACCgAgAA-5'
SNAIL	Human	ACTgCAACAAggAATACCTCAg	gCACTggTACTTCTTgACATCTg
SLUG	Human	TgTgACAAggAATATgTgAgCC	TgAgCCCTCAgATTTgACCTg
**18s**	Human	5'-CACCAACATCgATgggCgg-3'	3'-CACACgTTCCACCTCATCCTCAg-5'

Supplementary Methods Table 2: Antibodies used for western blot analysis (WB) and Immunoprecipitation (IP).

Antibody	Catalog Number	LOT Number	Company	Dilution
**GAPDH**	4300	01062548	Fisher	1:20,000
**pEGFR (Y1068)**	2236	18	Cell Signaling Technology	1:1000
**EGFR**	2239	5	Cell Signaling Technology	1:1000
**Cleaved PARP**	5625	18	Cell Signaling Technology	1:1000
**c-MYC**	ab32072	1053331-27	Abcam	1:1000
**CIP2A**	80659	H2522	Santa Cruz Biotechnology	1:1000
**E-cadherin**	14472S	8	Cell Signaling Technology	1:1000
**pS6 (S240/244)**	5364	13	Cell Signaling Technology	1:1000
**S6**	2317	13	Cell Signaling Technology	1:1000
**Zeb1**	701512T	2	Cell Signaling Technology	1:1000
**B55α**	5689S	3	Cell Signaling Technology	1:1000
**PP2A-C**	2038S	3	Cell Signaling Technology	1:1000
**PP2A-A**	2041S	2	Cell Signaling Technology	1:1000
**Normal Mouse IgG (IP)**	Sc-2025		Santa Cruz Technology	
**HA (IP & WB)**	2367S	5	Cell Signaling Technology	1:1000
**HA**	3724S	13	Cell Signaling Technology	1:1000
**Goat anti-Mouse IgG Secondary Antibody**	NC9401841	D20802-25	LICOR	1:5000
**Goat anti-Rabbit IgG Secondary Antibody**	NC0252291	D20809-05	LICOR	1:5000

Supplemental Data Methods 3: Antibodies used for immunofluorescence *in vitro* and tissue analysis.

Antibody	Catalog Number	LOT Number	Company	Dilution
Mouse E-cadherin	610181	3138351	BD Biosciences	1:200
Rabbit Vimentin	5741S	8	Cell Signaling Technology	1:200
Rhodamine Phalloidin	R415	2641902	Invitrogen	1:200 (*in vitro*)
Ku-80	2180S	3	Cell Signaling Technology	1:600 (tissue)
Goat anti-Rabbit IgG (H+L) Alexa Fluor™ Plus 594	A32740	VA295503	Invitrogen	1:2000 (*in vitro*) 1:1000 (tissue)
Goat anti-Mouse IgG (H+L) Alexa Fluor™ Plus 594	A32742	VA295504	Invitrogen	1:2000 (*in vitro*) 1:1000 (tissue)
Goat anti-Rabbit IgG (H+L) Alexa Fluor™ Plus 488	A32731	VA295501	Invitrogen	1:2000 (*in vitro*) 1:1000 (tissue)
Goat anti-Mouse IgG (H+L) Alexa Fluor™ Plus 488	A32723	VA297822	Invitrogen	1:2000 (*in vitro*) 1:1000 (tissue)
Goat anti-Rabbit IgG (H+L) Alexa Fluor™ Plus 647	A32733	VB296618	Invitrogen	1:2000 (*in vitro*) 1:1000 (tissue)
Goat anti-Mouse IgG (H+L) Alexa Fluor™ Plus 647	A32728	UK290265	Invitrogen	1:2000 (*in vitro*) 1:1000 (tissue)
